# MDITRE: Scalable and Interpretable Machine Learning for Predicting Host Status from Temporal Microbiome Dynamics

**DOI:** 10.1128/msystems.00132-22

**Published:** 2022-09-07

**Authors:** Venkata Suhas Maringanti, Vanni Bucci, Georg K. Gerber

**Affiliations:** a Department of Computer and Information Science, University of Massachusetts Dartmouth, Massachusetts, USA; b Department of Microbiology and Physiological Systems, University of Massachusetts, Medical School, Worcester, Massachusetts, USA; c Program in Microbiome Dynamics, University of Massachusetts Medical School, Worcester, Massachusetts, USA; d Department of Pathology, Brigham and Women’s Hospital, Boston, Massachusetts, USA; e Harvard Medical School, Boston, Massachusetts, USA; f MIT-Harvard Health Sciences and Technology, Cambridge, Massachusetts, USA; University of California San Diego

**Keywords:** artificial intelligence, host status, interpretable, machine learning, microbiome, time-series

## Abstract

Longitudinal microbiome data sets are being generated with increasing regularity, and there is broad recognition that these studies are critical for unlocking the mechanisms through which the microbiome impacts human health and disease. However, there is a dearth of computational tools for analyzing microbiome time-series data. To address this gap, we developed an open-source software package, Microbiome Differentiable Interpretable Temporal Rule Engine (MDITRE), which implements a new highly efficient method leveraging deep-learning technologies to derive human-interpretable rules that predict host status from longitudinal microbiome data. Using semi-synthetic and a large compendium of publicly available 16S rRNA amplicon and metagenomics sequencing data sets, we demonstrate that in almost all cases, MDITRE performs on par with or better than popular uninterpretable machine learning methods, and orders-of-magnitude faster than the prior interpretable technique. MDITRE also provides a graphical user interface, which we show through case studies can be used to derive biologically meaningful interpretations linking patterns of microbiome changes over time with host phenotypes.

**IMPORTANCE** The human microbiome, or collection of microbes living on and within us, changes over time. Linking these changes to the status of the human host is crucial to understanding how the microbiome influences a variety of human diseases. Due to the large scale and complexity of microbiome data, computational methods are essential. Existing computational methods for linking changes in the microbiome to the status of the human host are either unable to scale to large and complex microbiome data sets or cannot produce human-interpretable outputs. We present a new computational method and software package that overcomes the limitations of previous methods, allowing researchers to analyze larger and more complex data sets while producing easily interpretable outputs. Our method has the potential to enable new insights into how changes in the microbiome over time maintain health or lead to disease in humans and facilitate the development of diagnostic tests based on the microbiome.

## INTRODUCTION

The human microbiome is highly temporally dynamic (1). Some of the most profound changes over time occur during infancy and early childhood, when the microbiome is first becoming established (2–4). Although the microbiome is more stable in adulthood, it continues to undergo significant changes over time due to diet (5, 6), travel (7), antibiotic use (8), infection (7), gut inflammation (9), and a variety of other factors. Microbial dynamics, particularly those early in life, have been linked to many human diseases, including necrotizing enterocolitis (10), diabetes (4, 11), food allergies (12), obesity (13), and inflammatory bowel diseases (9). An increasing number of prospective longitudinal studies have been undertaken to characterize microbiome-disease relationships. Such longitudinal studies are particularly important for advancing the field because they can help establish causality (e.g., changes definitively preceding disease onset) and provide information for clinically useful diagnostic or prognostic tests.

Relatively few computational or statistical methods have been specifically developed to analyze longitudinal microbiome data, despite its importance to the field. Human microbiome time-series data present numerous challenges, including small numbers of subjects, high subject-to-subject variability, case/control imbalance, irregular/sparse temporal sampling, high-dimensionality, compositionality, and complex dependencies among variables (1). Methods that have been developed for analyzing microbiome time-series data generally fall into four categories: (i) univariate models of taxa trajectories (for example, see Joseph et al. [14]), which are useful for interpolating data or characterizing differences over time between two cohorts on a taxon-by-taxon basis; (ii) dynamical systems models that capture microbe-microbe interactions (15, 16), which are useful for forecasting ecosystem behaviors over time such as responses to perturbations or stability; (iii) unsupervised learning or clustering methods, which are useful for characterizing common patterns of change among microbes (5); and (iv) supervised learning methods that predict host status or outcomes using trajectories of multiple taxa as inputs (17, 18), which are useful for establishing associations between microbiome dynamics and host phenotypes or developing diagnostic/prognostic tests. Our present work falls into the latter category.

In the supervised learning domain, general-purpose “black-box” machine learning methods, such as deep neural networks and random forests, have become increasingly popular. Indeed, such methods have been applied to microbiome data and have been demonstrated to accurately predict host phenotype (19, 20), including from longitudinal data (18). Although these methods can achieve high predictive performance, by their nature they encode mathematical functions which are incomprehensible to humans. In some domains, such as speech recognition for consumer applications, human comprehension of the underlying model is not important. However, in many biomedical domains, including the microbiome, understanding the underlying model is critical; the end-consumers of analyses are often wet-lab experimentalists or clinicians, who ultimately seek to generate specific testable hypotheses or develop diagnostic/prognostic clinical tests. One approach for understanding black-box models is *post hoc* techniques that attempt to explain individual components of black-box models with simpler models. For example, in the microbiome domain, the Local Interpretable Model Agnostic Explanations (LIME) (21) technique has been applied to random forests to attempt to find the abundance thresholds of specific microbes that differentiate patients according to disease severity (22). Although these *post hoc* techniques are useful, they suffer from several limitations, including inherent unfaithfulness to the original model or difficulty expressing how inputs are jointly related to each other (23, 24).

Some alternatives to black-box machine learning methods are models that are purposefully constructed to be interpretable. The notion of interpretability is inherently domain-specific and ultimately hinges on the ability of human experts to comprehend the models (23). In prior work, we introduced the Microbiome Interpretable Temporal Rule Engine (MITRE) (17), a fully Bayesian, microbiome time-series-specific model that learns human-interpretable rules to classify the host’s status (e.g., healthy or diseased) from microbiome time-series data. MITRE rules consist of conjunctions of *detectors* that handle dependencies in both microbiome and time-series data. These detectors are conditional clauses of the form: “*TRUE* if the aggregated abundance (or rate of change of abundances) of microbes in phylogenetic subtree *A* within time window *T* is above threshold *B*.” This approach, which performs a set of nonlinear but interpretable and domain-specific transformations on the inputs, was shown to perform on par with, and/or outperform, black-box machine learning methods (i.e., random forests). However, because MITRE uses a sampling-based inference approach, which operates combinatorically on a large space of pre-computed features, it is not scalable to increasingly large microbiome data sets.

One exciting recent direction is leveraging advances in computing technologies originally developed for black-box deep learning to greatly accelerate interpretable logic or rule-based approaches. At the core of the deep learning revolution are software and hardware advances, including graphical processing units (GPUs) that perform highly parallelized computations to optimize nonlinear functions using gradient descent-based methods. These approaches require that the functions to be optimized are differentiable. Standard logic or rule-based models are not differentiable because logical clauses and their combinations are discrete, not continuous, entities. To tackle this problem, relaxation approaches can be used, which construct smooth approximations to the underlying logical functions that are successively made sharper throughout the learning algorithm (25–27).

Building on this work, to achieve scalability on large microbiome data sets while maintaining model interpretability, we developed the Microbiome Differentiable Interpretable Temporal Rule Engine (MDITRE), a fully differentiable version of our original MITRE method. The remainder of the manuscript is organized as follows. First, we introduce the MDITRE model, including domain-specific microbiome- and temporal-focusing mechanisms, which enable model differentiability. We also provide details on the MDITRE open-source software package, which can run on GPUs and provides a graphical user interface. Next, we present predictive performance and run-time benchmarking results of MDITRE against MITRE and other methods, on both semi-synthetic and real microbiome data sets, which study a variety of host phenotypes/outcomes. We also show that MDITRE can scale to much larger data sets than MITRE can feasibly run on, through both semi-synthetic and real data. Finally, we provide cases studies illustrating MDITRE’s ability to readily uncover biologically interpretable patterns in data sets using its automatically inferred rules and visualization capabilities.

## RESULTS

### MDITRE has a fully differentiable architecture that enables scalability while maintaining interpretability.

**FIG 1:**
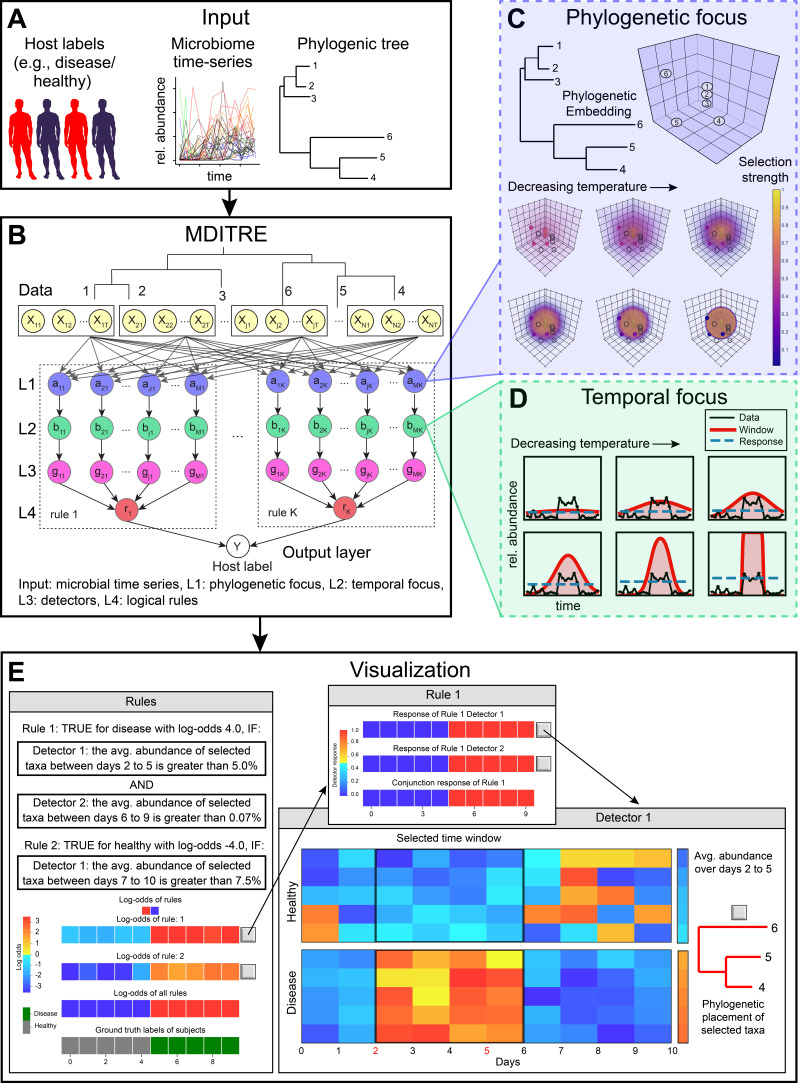
Microbiome Differentiable Interpretable Temporal Rule Engine (MDITRE) efficiently learns rule-based machine learning models that predict host status from microbiome time-series data. (A) The inputs to MDITRE consist of binary labels for subjects (e.g., diseased versus healthy), relative abundances of microbiomes over time (derived from either 16S rRNA gene amplicon or metagenomics sequencing), and a phylogenetic tree. (B) MDITRE efficiently learns human-interpretable rules from the input data, using continuous relaxation techniques that render the model fully differentiable and amenable to deep-learning optimization techniques. The model consists of a five-layer neural network, which includes specialized layers to learn phylogenetic and temporal features and combine these features into rules. (C) Schematic of phylogenetic focus nodes, which embed taxa based on their phylogenetic distances and learn groups of related taxa to be used in rules. With decreasing temperature, the groups become sharper. (D) Schematic of temporal focus nodes, which learn relevant time windows to be used in rules. With decreasing temperature, time windows become sharper. (E) MDITRE output is a set of human-readable rules. Each rule consists of a conjunction (“AND”) of detectors that each selects a relevant set of taxa, a time window, and a threshold (for either aggregated abundance or rate-of-change of abundance). A graphical interface allows users to view per-subject strengths of rules (log-odds) and visualizations of detector activations and components.

MDITRE is a highly scalable approximation to our previous MITRE method (17), a fully Bayesian supervised machine learning framework that classifies hosts according to specified binary labels (e.g., diseased or healthy) using microbiome time-series data. MDITRE takes as input (Fig. 1A): (i) a binary (two-value) label of the status of each host, (ii) a table of microbial longitudinal relative abundances (operational taxonomic units [OTUs] or amplicon sequence variants [ASVs] from 16S rRNA sequencing, or taxa derived from shotgun metagenomic data), and (iii) a table of pairwise distances (matrix of phylogenetic distances among taxa). Using these data as inputs, MDITRE learns human-interpretable *rules*, which explicitly incorporate microbiome- and temporal- specific features, to output predictions of host labels (Fig. 1B and E). A rule consists of a conjunction (logical AND) of *detectors*. Detectors are of the form “*TRUE* if the [aggregated abundance/rate of change of abundance] of taxa in group *A* within time window *T* is above threshold *Y.*” The label for each host (e.g., healthy or diseased) is then predicted by the model based on a weighted sum of rules; the weights on the rules can be interpreted as the odds of predicting a particular host label given the input microbiome time-series information.

To create MDITRE, we introduced a set of relaxations, or continuous approximations, to discrete variables in the original MITRE model. These approximations render the MDITRE model fully differentiable, and thus amenable to highly parallel hardware-accelerated learning. Our original MITRE method effectively enumerated all subsets (phylogenetic subtrees) of microbes, time-windows, and abundance thresholds and then used a sampling-based inference algorithm to probabilistically explore this high-dimensional combinatoric space. MDITRE does away with explicit enumeration of features and instead directly (and continuously) parameterizes the model space. We accomplished this through several modeling innovations, including what we term microbiome or temporal *group focus* functions (Fig. 1C and D), which perform “soft” selections over sets of microbes or time points. To incorporate prior biological information into the microbe features, in terms of phylogenetic relationships, we introduced an embedding in phylogenetic space that “anchors” group focus functions. We also employed a relaxation of the logical AND operation, which was inspired by Neural Arithmetic Units (27). As with our previous fully Bayesian model, we placed prior probability distributions on variables in the MDITRE model to incorporate biological information or to encourage model sparsity (i.e., total number of detectors or rules). We used relaxed versions of probability distributions to maintain differentiability. See Methods and Supplemental Methods for complete details.

### MDITRE was implemented in an open-source software package that uses standard deep learning libraries and has a graphical user interface.

MDITRE can be represented as a five-layer neural network (Fig. 1B), which allows us to directly leverage standard deep learning software packages. The top-most layer (layer 1) performs phylogenetic focus, generating outputs that are aggregated abundances of bacteria within the phylogenetically focused regions (Fig. 1C). The temporal focus layer (layer 2) computes the average (or the rate of change) of its input (phylogenetically focused abundances) over temporally focused time windows (Fig. 1D**)**. The detector layer (layer 3) computes “soft” binary detector activations based on its inputs and detector thresholds. The rule layer (layer 4) performs “soft AND” operations over the input detector activations and then sends rule activations on to the last layer. Finally, the classification layer (layer 5) aggregates the rule activations from the previous layer to predict host labels.

We implemented MDITRE in Python using the PyTorch (28) deep learning library, which fully supports GPU hardware acceleration, and have made the software package available under an open-source license. For model learning, we use standard gradient-descent based approaches in PyTorch to perform maximum *a posteriori* estimation of model parameters (see Methods and Supplemental Methods for complete details). In addition to learning the model, the software provides a graphical user interface for visualizations of the learned rules, allowing end-users to readily interpret outputs (Fig. 1E); we provide illustrative examples of these visualizations through the use-case scenarios described below. We also provide a tutorial, which guides users step-by-step from data set processing to final interpretations of outputs, to facilitate ease-of-use (see Materials and Methods).

### MDITRE performed comparably to our previous method MITRE but with up to orders-of-magnitude faster run-times.

We first benchmarked MDITRE’s predictive performance on semi-synthetic time-series data sets generated using MITRE’s data simulation procedure. Briefly, simulated data were generated from real data using a parametric bootstrapping-type procedure, and perturbations of one or two randomly chosen microbial clades were added to subsets of artificial subjects to simulate diseased/dysbiotic states. Simulations were run for different numbers of artificial subjects, ranging from 20 to 1,024, and for different numbers of time points, ranging from 6 to 30 (for the case corresponding to 32 subjects). These ranges were chosen to correspond to sizes of real data sets and to test the scalability of the methods to larger data sets. As with our previous comparisons (17), we also benchmarked against L1 regularized logistic regression (L1) and random forest (RF), which are an interpretable linear method and a black-box nonlinear method, respectively.

Model performance was estimated using a 5-fold cross-validation procedure for model selection followed by validation on an independently generated test data set of the same size as the training data set. Variability of performance was quantified using 10 simulations (i.e., 10 different random seeds) of each synthetic data set, and 10 runs (i.e., 10 different random seeds) of each algorithm over each data set. As metrics for comparison, we used both F1-scores (harmonic mean of precision and recall) and area under the curve (AUC) of receiver operating characteristic curves.

**FIG 2:**
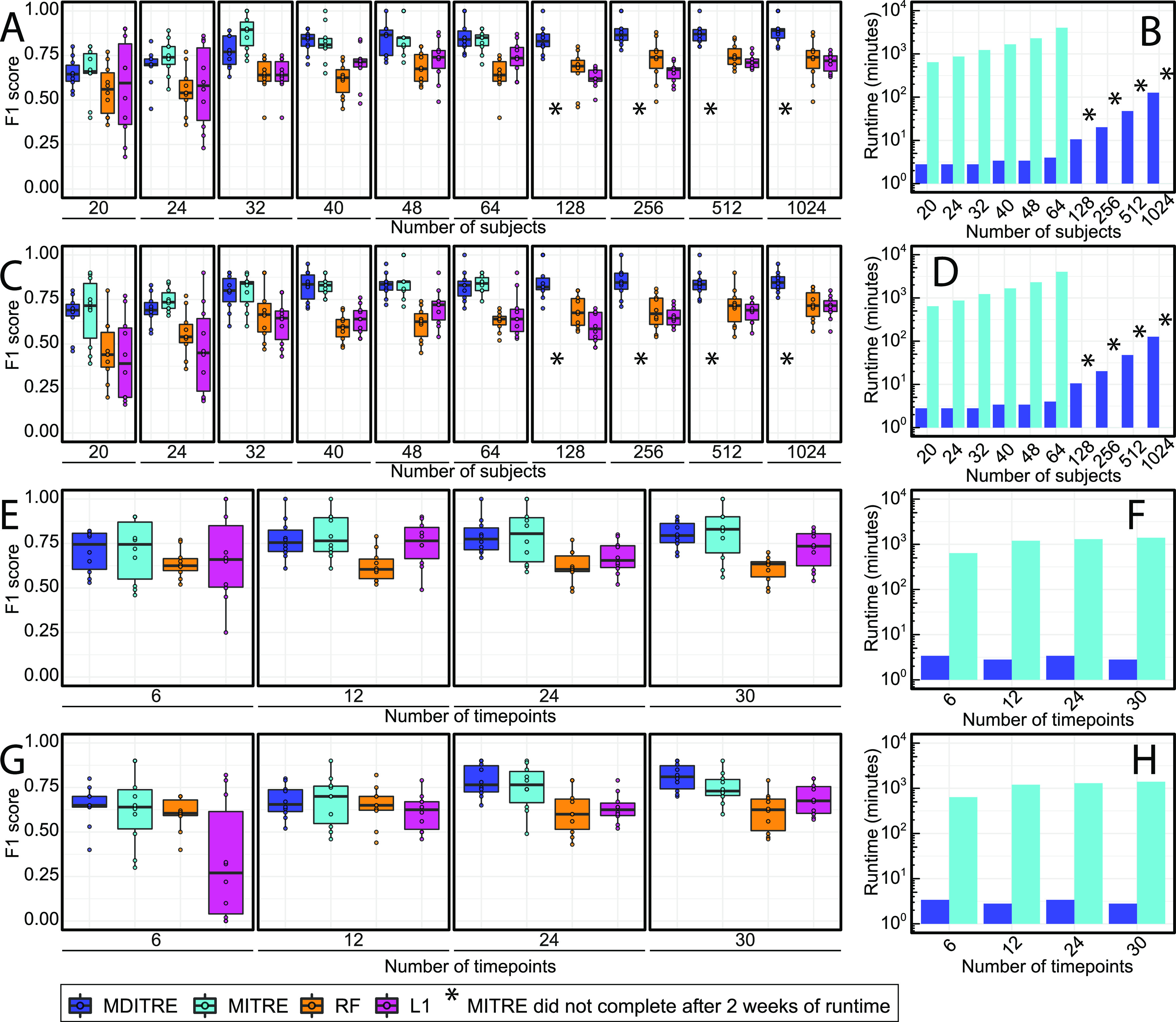
MDITRE outperformed random forests and performed comparably to our previous interpretable method with up to orders of magnitude faster run-times in almost all cases on synthetic data. Classification performance for all methods was assessed using a 5-fold cross-validation procedure for model selection followed by validation on an independently generated held-out test data set of the same size as the training data set. This process was repeated 10 times, each corresponding to a different random seed. (A, C, E, and G) Performance in terms of F1 scores (harmonic mean of precision and recall): (A) one or (C) two microbial clades perturbed with increasing numbers of subjects with 18 time points, (E) one or (G) two microbial clades perturbed with increasing numbers of time points and 32 subjects. Panels B, D, F, and H show corresponding runtimes (in log_10_ minutes). (I) Performance on real data. (J) Corresponding runtimes. RF, random forest; L1, L1 regularized logistic regression; Bok, Bokulich; del, delivery. For the F1 score comparison, hypothesis testing was performed using the Mann-Whitney U test and a significance threshold of 0.05. For boxplots: solid line indicates the median, lower and upper hinges correspond to the first and third quartiles (25th and 75th percentiles), and upper whisker extends from the hinge to the largest value no further than 1.5 × IQR (interquartile range, or distance between the first and third quartiles) from the hinge. The lower whisker extends from the hinge to the smallest value at most 1.5 × IQR from the hinge.

For the F1 score, MDITRE almost always performed comparably to MITRE, and outperformed L1 and RF (Fig. 2A, C, E, and G). For the case with one simulated perturbation and increasing numbers of subjects (Fig. 2A), MDITRE performed comparably to MITRE in every case (*P* > 0.05, Mann-Whitney U test; see Data Set S1, tab 1 in the supplemental material), excluding the simulations with 32 subjects, in which MITRE outperformed MDITRE (8% lower average performance for MDITRE). Moreover, MDITRE also achieved better performance than L1 and RF in every case (*P* < 0.05), excluding the simulations with 20 (where L1 and RF performed comparably to MDITRE) or 24 subjects (where L1 performed comparably to MDITRE). For the case with two simulated perturbations and increasing numbers of subjects (Fig. 2C), MDITRE performed comparably to MITRE (*P* > 0.05; Data Set S1, tab 2) and outperformed L1 and RF methods in all cases (*P* < 0.05). Evaluation using the AUC metric and Delong’s method for statistical testing (29) produced analogous results (Fig. S1 and Data Set S1, tabs 5 to 8), with MDITRE outperforming RF and L1 and performing comparably to MITRE in all cases with increasing numbers of subjects and most cases with increasing numbers of time points (Fig. S1, Data Set S1, tabs 5 to 8).

10.1128/msystems.00132-22.1 FIG S1 Classification performance on semi-synthetic data in terms of area under the curve (AUC) and assessment of statistical significance with Delong’s test. (A to D) Classification performance for all methods was assessed using a 5-fold cross-validation procedure for model selection, followed by validation on an independently generated held-out test dataset of the same size as the training dataset. This process was repeated 10 times, each corresponding to a different random seed. For the boxplots, the solid line indicates the median AUC, the lower and upper hinges correspond to the first and third quartiles (25th and 75th percentiles), and the upper whisker extends from the hinge to the largest value no further than 1.5 × IQR (interquartile range, or distance between the first and third quartiles) from the hinge. The lower whisker extends from the hinge to the smallest value at most 1.5 × IQR from the hinge. (E to H) Distribution of *P* values using Delong’s method. Testing was performed relative to Microbiome Differentiable Interpretable Temporal Rule Engine (MDITRE)’s performance for each data-point; thus, smaller *P* values indicate larger differences in performance between MDITRE and the comparator method. Dashed line corresponds to a *P* value of 0.05. Download FIG S1, TIF file, 2.0 MB. Copyright © 2022 Maringanti et al. 2022 Maringanti et al. https://creativecommons.org/licenses/by/4.0/ This content is distributed under the terms of the Creative Commons Attribution 4.0 International license.

10.1128/msystems.00132-22.7 DATA SET S1 Results from the statistical testing on models’ performance on semi-synthetic and real data, and results from models’ runtime comparison. Download Data Set S1, XLSX file, 0.2 MB. Copyright © 2022 Maringanti et al. 2022 Maringanti et al. https://creativecommons.org/licenses/by/4.0/ This content is distributed under the terms of the Creative Commons Attribution 4.0 International license.

Overall, as we have previously reported (17), for increasing numbers of subjects, we noticed a general increase in performance for all of the methods, which eventually plateaued on cases with >48 subjects. For all cases with increasing numbers of time points (Fig. 2E and G), MDITRE achieved comparable performance to MITRE (*P* > 0.05; Data Set S1, tabs 3 to 4). MDITRE outperformed L1 and RF in most cases with increasing numbers of time points, except for a few cases in which the methods performed comparably (Data Set S1, tabs 3 to 4). Similar to what we reported for MITRE in our previous work (17), for the cases with one simulated perturbation, we found no significant increase in performance with increasing numbers of time points per subject, while for the case with two simulated perturbations, we found only a slight increase in performance with increasing numbers of time points. Both trends could be explained by the fact that sampling additional time points that can lie outside the perturbation windows of interest provides no additional information to the model while potentially adding noise that makes prediction more challenging.

To further assess the abilities of the different methods to generalize on unseen data, we generated a series of independent test data sets with increasing levels of measurement noise relative to the training data set (1×, 10×, 100×, 1,000×, and 10,000×). This scenario simulates studies with the same underlying biological signal and variability but different measurement noise characteristics, such as could occur if the samples were processed in different labs. Paralleling the simulated data experiments described above, we used MITRE’s synthetic data generation model and evaluated cases with either one or two external perturbations. As expected, we saw degradations in performances for all methods with increasing noise levels (see Fig. S2 for F1 score and Fig. S3 for AUC), with performance reduced by ~50% at the highest noise level. In addition, we saw decreased performance with the more challenging two-perturbation case and in cases with lower numbers of subjects. However, all methods showed some degree of generalization capability, and MDITRE still performed comparably to MITRE and outperformed RF and L1 in all the cases (see Data Set S1, tabs 9 and 10 for F1 scores; Fig. S4 and Data Set S1, tabs 11 and 12 for AUCs), except for the cases with noise levels of ≥1,000×, in which no method performed effectively. These results suggest that all of the methods could generalize to an extent in this scenario with increasing measurement noise, but that MITRE and MDITRE consistently outperformed the comparator methods even at high noise levels.

10.1128/msystems.00132-22.2 FIG S2 Classification performance on semi-synthetic data with increasing levels of simulated measurement noise in terms of F1 scores. Models were tested on independently generated data sets with increasing levels of simulated measurement noise relative to the training data (1×, 10×, 100×, 1,000×, 10,000×). Download FIG S2, TIF file, 1.2 MB. Copyright © 2022 Maringanti et al. 2022 Maringanti et al. https://creativecommons.org/licenses/by/4.0/ This content is distributed under the terms of the Creative Commons Attribution 4.0 International license.

10.1128/msystems.00132-22.3 FIG S3 Classification performance on semi-synthetic data with increasing levels of simulated measurement noise in terms of AUC. Models were tested on independently generated data sets with increasing levels of simulated measurement noise relative to the training data (1×, 10×, 100×, 1,000×, 10,000×). Download FIG S3, TIF file, 1.2 MB. Copyright © 2022 Maringanti et al. 2022 Maringanti et al. https://creativecommons.org/licenses/by/4.0/ This content is distributed under the terms of the Creative Commons Attribution 4.0 International license.

10.1128/msystems.00132-22.4 FIG S4 Statistical testing of classification performance with increasing levels of simulated measurement noise on semi-synthetic data in terms of AUC. Distribution of *P* values using Delong’s method is shown. Testing was performed relative to MDITRE’s performance for each data-point; thus, smaller *P* values indicate larger differences in performance between MDITRE and the comparator method. Dashed line corresponds to a *P* value of 0.05. Download FIG S4, TIF file, 1.3 MB. Copyright © 2022 Maringanti et al. 2022 Maringanti et al. https://creativecommons.org/licenses/by/4.0/ This content is distributed under the terms of the Creative Commons Attribution 4.0 International license.

In terms of runtime, MDITRE achieved significant speedups over MITRE in all cases on semi-synthetic data (Fig. 2D, F, and H and Data Set S1, tab 13), with particularly impressive speedups of >1,000× on cases with larger numbers of subjects. Further, we were unable to complete benchmarking of MITRE for 128 subjects or greater because in these cases, MITRE still had not completed runs after 2 weeks of compute-time on our cluster; in contrast, MDITRE ran on the case with the largest number of subjects, 1,024, in approximately 2 hours. This huge speedup in MDITRE’s runtime is attributable to its fully differentiable architecture, which enabled us to implement an efficient gradient-descent-based learning algorithm, in contrast to the much slower Markov Chain Monte Carlo (MCMC)-based learning algorithm employed by MITRE.

We next benchmarked MDITRE’s predictive performance on eight classification tasks from seven published human microbiome data sets: (i) Bokulich et al. (2), a study of gut microbiomes of 37 infants sampled over the first 2 years of life, with two separate classifications, diet (breastfed versus formula) and birth mode (vaginal versus C-section); (ii) David et al. (6), a study of microbiomes of 20 healthy adults receiving dietary interventions (animal- versus plant-based); (iii) DiGiulio et al. (30), a study of vaginal microbiomes of 37 pregnant women (at-term versus preterm delivery); (iv) Vatanen et al. (31), a study of gut microbiomes of 117 children sampled over the first 3 years of life (Russian versus Estonian/Finnish nationality); (v) Kostic et al. (4), a study of gut microbiomes of 17 infants sampled over the first 3 years of life (normal versus development of type 1 diabetes); (vi) Brooks et al. (32), a study of gut microbiomes of 30 infants sampled over 75 days (vaginal versus C-section); and (vii) Shao et al. (33), a study of gut microbiomes of 282 infants (after filtering for subjects with fewer than three time points) sampled over 424 days (vaginal versus C-section). Datasets i-iv consist of 16S rRNA amplicon sequencing data, and datasets v-vii consist of shotgun metagenomics data. See Methods for a complete description of bioinformatics and preprocessing of data sets. To estimate performance for the first six data sets, which have relatively small numbers of subjects, we used repeated 5-fold cross-validation. In this procedure, which provides greater robustness, cross-validation is performed multiple times, with the folds split in a different way for each repetition (as opposed to non-repeated cross-validation, which performs only a single partitioning of the data set). For our analysis, we used 5 repetitions and 10 random seeds within each repetition. However, any form of cross-validation suffers from limitations in estimating true generalization performance. The Shao et al. data set, consisting of >200 subjects, gave us the opportunity to do a true hold-out performance assessment; for this data set, we completely held-out 25% of the data (randomly selected) as a test set and trained on the remaining data.

**FIG 3:**
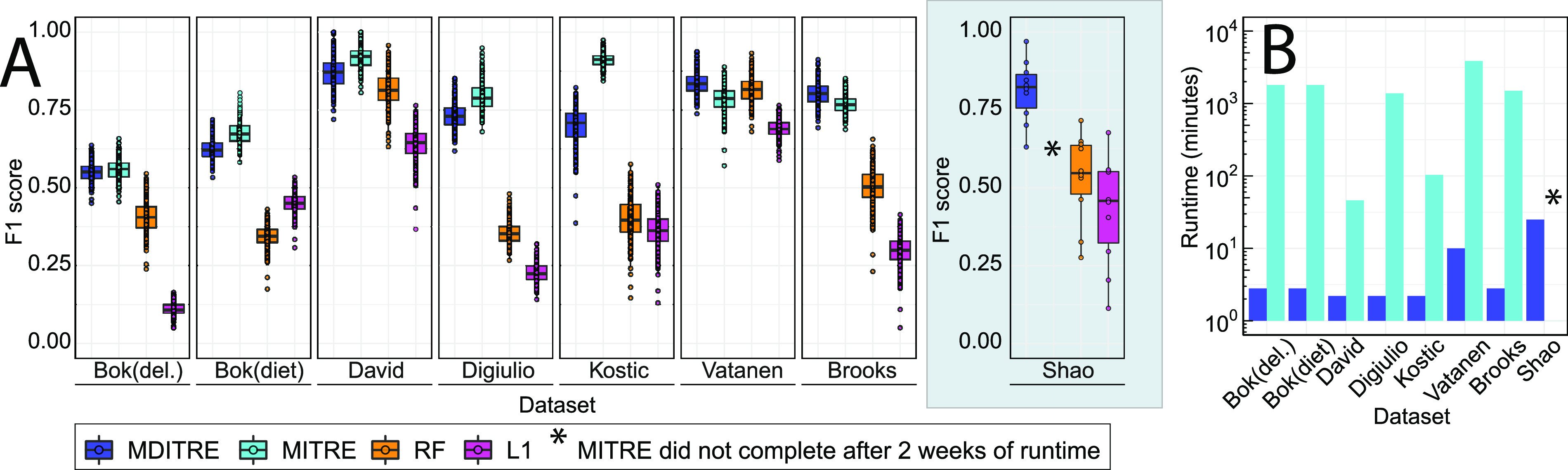
MDITRE outperformed random forests and performed comparably to our previous interpretable method with up to orders of magnitude faster run-times in almost all cases on real data. Classification performance on all data sets except for Shao et al. was assessed using 5-fold repeated cross-validation with five repetitions (each a different random partition of the data). This procedure was run 10 times, each time using a different random seed, which resulted in a total of 50 performance evaluation scores. For the Shao et al. data set (gray box), we assessed performance on a held-out test set consisting of 25% of the data (randomly selected), with the remaining 75% used solely for training and model selection. (A) Performance on real data and (B) corresponding runtimes. For the F1 score comparison, hypothesis testing was performed using the Mann-Whitney U test and a significance threshold of 0.05. For boxplots: solid line indicates the median, lower and upper hinges correspond to the first and third quartiles (25th and 75th percentiles), and the upper whisker extends from the hinge to the largest value no further than 1.5 × IQR from the hinge. The lower whisker extends from the hinge to the smallest value at most 1.5 × IQR from the hinge.

On real data, using the F1 score as the metric, MDITRE performed comparably to MITRE and outperformed the L1 and RF methods in most cases (Fig. 3A and Data Set S1, Tab 14), while achieving massive speedups over MITRE (Fig. 3B). Specifically, MDITRE had comparable performance to MITRE on six of the eight classification tasks (*P* > 0.05; Mann-Whitney U Test), while underperforming MITRE on the DiGiulio et al. and Kostic et al. data sets (9% and 15% lower average performance, respectively) (Data Set S1, tab 14). MDITRE significantly outperformed the L1 and RF methods on all data sets (*P* values < 0.05, Data Set S1, tab 14). In terms of AUCs, MDITRE achieved comparable performance to MITRE on all data sets except for Kostic et al., where MDITRE slightly underperformed MITRE (7% lower AUC for MDITRE) (Fig. S5; Data Set S1, tab 15). Moreover, MDITRE outperformed RF and L1 in terms of AUC on all cases apart from Vatanen et al. (comparable performance to RF) and David et al. (comparable performance to RF).

10.1128/msystems.00132-22.5 FIG S5 Classification performance on real data and statistical testing in terms of AUC. Performance on all data sets except for Shao et al was assessed using five-fold repeated cross-validation with five repetitions (each a different random partition of the data). This procedure was run 10 times, each time using a different random seed, which resulted in a total of 50 performance evaluation scores. For the Shao et al data set (gray box), we assessed performance on a held-out test set consisting of 25% of the data (randomly selected), with the remaining 75% used solely for training and model selection. (A) Classification performance reported as AUC. (B) Distribution of *P* values using Delong’s method. Testing was performed relative to MDITRE’s performance for each data-point; thus, smaller *P* values indicate larger differences in performance between MDITRE and the comparator method. Dashed line corresponds to a *P* value of 0.05. Download FIG S5, TIF file, 0.8 MB. Copyright © 2022 Maringanti et al. 2022 Maringanti et al. https://creativecommons.org/licenses/by/4.0/ This content is distributed under the terms of the Creative Commons Attribution 4.0 International license.

In terms of runtimes (Fig. 3B and Data Set S1, tab 13), MDITRE achieved speedups over MITRE ranging from 86× to 1,150×. MITRE was unable to run on the Shao et al. data set due to its size, whereas MDITRE completed analysis of this data set in 24 min. We note that for the cases in which MDITRE underperformed MITRE, these were the most imbalanced or small data sets. The DiGiulio et al. data set is highly imbalanced, with only 6 of 37 subjects belonging to the “positive” group, and Kostic et al. is the smallest data set, with 17 subjects. Thus, predictive accuracy results for these data sets should be interpreted with caution, as high sample imbalance or small sample size could lead to less reliable estimates for cross-validated performance measures. Overall, our results on both semi-synthetic and real data sets demonstrate that MDITRE, which uses continuous relaxations to approximate discrete variables in our MITRE model, achieves competitive classification performance to our original method, while running orders of magnitude faster and scaling to much larger data sets.

### MDITRE discovered human interpretable rules that automatically focused on biologically relevant taxa and time windows.

We next examined the interpretability of MDITRE’s outputs, which we demonstrate through two case studies. In the previous section, we focused on objective measures of predictive performance. However, for many microbiome applications, the critical tasks are discovering relationships between the microbiome and host and finding clinically useful biomarkers, rather than pure prediction. For these purposes, model interpretability, which is inherently domain-specific and subjective (23), is the key property of interest. Through the case studies in this section, we illustrate powerful features of the rules and visualizations that MDITRE returns, which facilitate interpretability tailored to the domain of microbiome time-series analyses through: (i) automatic focus on relevant groups of taxa or single taxa that differentiate hosts, (ii) automatic focus on time windows in which the microbiome is differentially changing depending on host status and, (iii) human-readable rules with “AND” and “OR” logic, which can capture rich patterns of dynamics or host variation, while remaining easily understandable.

**FIG 4:**
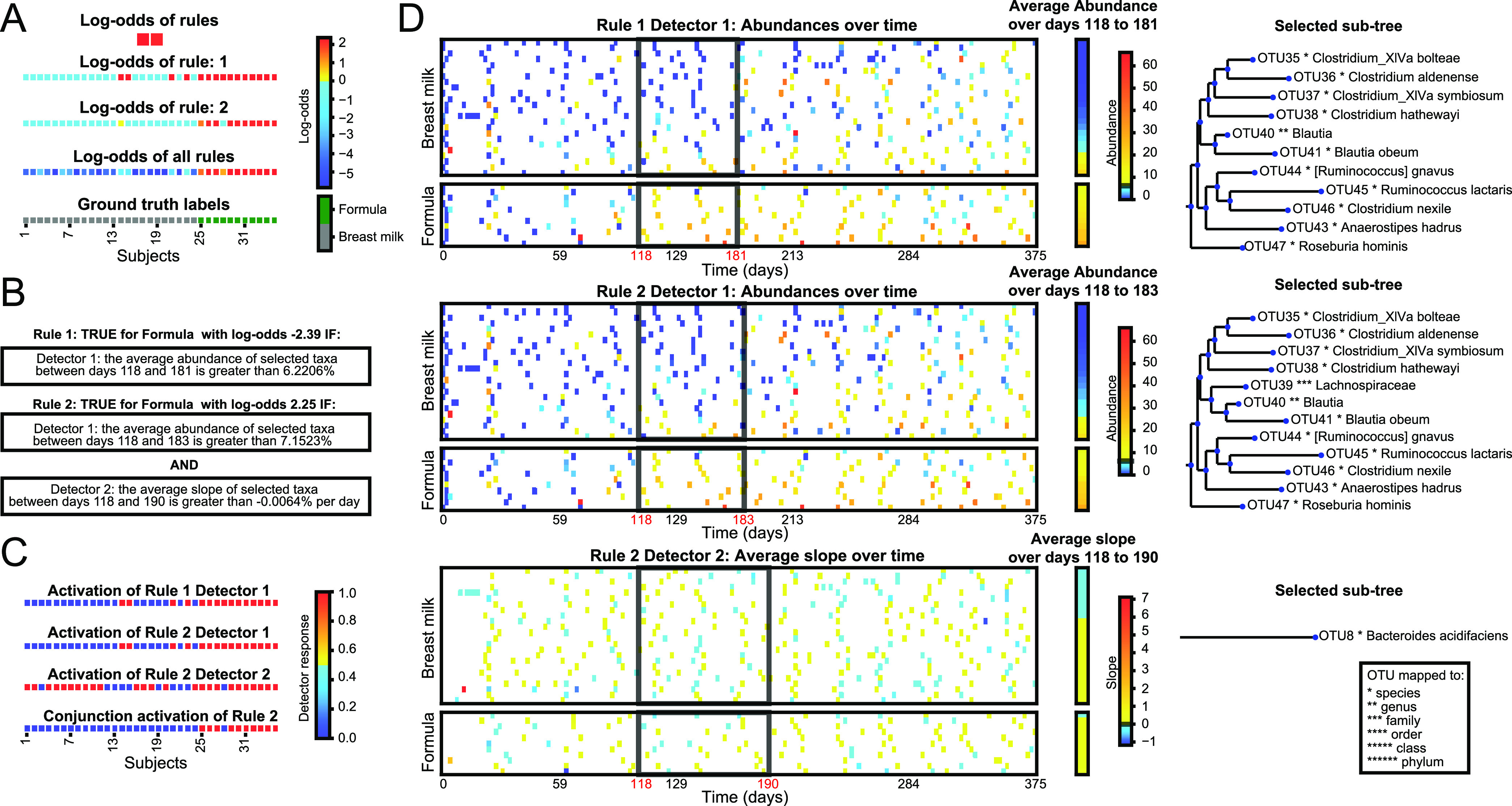
Case study one: MDITRE rules which distinguish predominantly breast versus formula-fed infants based on gut microbiome changes over time. Visualizations output by the MDITRE graphical user interface are shown for analysis of data from Bokulich et al., which tracked the gut microbiomes of 37 infants using 16S rRNA gene amplicon sequencing. (A) MDITRE identified two rules which, in combination, accurately differentiate infants who were predominantly breast versus formula-fed. Colors indicate the degree to which each rule, or the rules in combination, contribute to the odds of predicting the status (i.e., formula or breast milk fed) of each infant. Yellow-to-red colors indicate that the infant is more likely to be formula-fed (with red indicating the highest likelihood); cyan-to-blue colors indicate the infant is more likely to be breast milk-fed (with blue indicating the highest likelihood). (B) Human-readable logic of the rule. (C) Per-subject activations (“truth” values) for each detector in each rule, and the conjunction (combined effects) of detectors for rules which have more than one detector. Colors indicate the degree to which each detector (clause) of the rule is true for each infant. Red indicates that the detector is true, blue indicates that the detector is false. (D) Visualization of per-subject data aggregated by each detector, as well as the detector’s time-window (black boxes), threshold for either abundance or slope (black line on abundance or slope legend), and selected taxa. Colors indicate the degree to which the average aggregated relative abundance (or rate of change in relative abundance) is above or below the detector threshold. Yellow-to-red indicates above the threshold (with red the highest value) and cyan-to-blue indicates below the threshold (with blue the lowest value).

**(i) Case study one: the relationship between diet and the microbiome in infants.** Our first case study used the data set of Bokulich et al., which analyzed gut microbiomes of 37 infants during the first 2 years of life, using 16S rRNA amplicon sequencing. Note that although there were samples over 2 years, they trailed off significantly after the first year of the study, and our preprocessing procedure to ensure sufficient samples in time-windows truncated the analyzable data to the first 375 days (see Materials and Methods and (17) for complete details). Given the task of classifying infants as receiving either breast milk or formula predominant diets, MDITRE learned two rules. Fig. 4 illustrates information available through MDITRE’s graphical user interface, which aids in interpreting these rules. Fig. 4A illustrates how the rules are combined in a logical “OR” to predict host labels. In this case, we can see that each rule alone can correctly classify most infants, but not necessarily with high odds. However, when the rules are combined, the separation between the classes is improved (i.e., higher odds of classifying as either formula or breastfed), and several infants that would be misclassified by individual rules were correctly classified by the combined rules.

By clicking on a rule, the user can then see an English description (Fig. 4B) and a visualization of how the rule’s detectors combine in a logical “AND” to determine the rule’s final truth value (Fig. 4C). For brevity, we discuss the second rule, which contains two detectors. The first detector is true for all the predominantly formula-fed infants, but also four of the breast-fed infants. In contrast, the second detector is true for more infants overall (both formula- and breast-fed) but is false for the four breast-fed infants the first detector was true for; this suggests the second rule finds complementary information to aid in defining breast-fed status for a group of infants. The conjunction of the detectors then provides a final rule that is true for all but one formula-fed infant, and false for all the breastfed infants.

By clicking on the detectors, the user can then view visualizations (Fig. 4D) of the time windows and taxa selected by each detector. Both detectors for the second rule focus on approximately the same time window, between about four to 6 months. Interestingly, this is a period directly preceding the introduction of the first solid foods for most infants (and which may occur earlier for formula-fed infants (34), thus explaining why MDITRE may have selected this time-period rather than a later one). Because infant microbiomes are extremely variable for the first few months of life (35), the automatically selected time window seems to reflect an optimal period for differentiating formula- versus breastfed infants: when the microbiome has had time to equilibrate, but before a new perturbation introduced by solid foods.

The first detector for the second rule, an aggregate abundance type, selected 12 taxa in the order Clostridiales, including the *Clostridium*, *Blautia*, *Ruminococcus*, *Anaerostipes*, and *Roseburia* genera. This detector, which is true when the aggregate abundance of these taxa is greater than ~7%, detects all the formula-fed infants and four of the breast-fed infants (because the study only reported the predominant feeding mode, it is possible these infants also received relatively more formula than others in the breastfed group). By focusing on the aggregated abundance of these taxa, the detector has automatically uncovered a group of phylogenetically related microbes that may not all be present in one individual or all in large amounts, but in aggregate may reflect a common biological function. Indeed, many of the selected taxa are strict anaerobes that metabolize more complex nutrient sources, including starches and lipids (36) that may be present in formula. Interestingly, the visualization produced by MDITRE suggests that after the selected time window, the abundance of these taxa became increasingly difficult to distinguish between formula- or breast-fed infants, which may reflect similar diets post-liquid-foods in both groups. This again highlights the ability of MDITRE to automatically focus on relevant time windows.

The second detector, a slope-type, selects a single taxon, Bacteroides acidifaciens, and is true if this taxon is increasing. This detector was not only true for all but one formula-fed infant but also many breast-fed infants; however, it was false for the four breastfed infants identified by the first detector. Bacteroides acidifaciens has been shown to increase with higher fiber diets (37). Thus, this detector may be capturing a gradient of the introduction of solid foods in the infants, with delayed introduction of solid foods for some breastfed infants.

**FIG 5:**
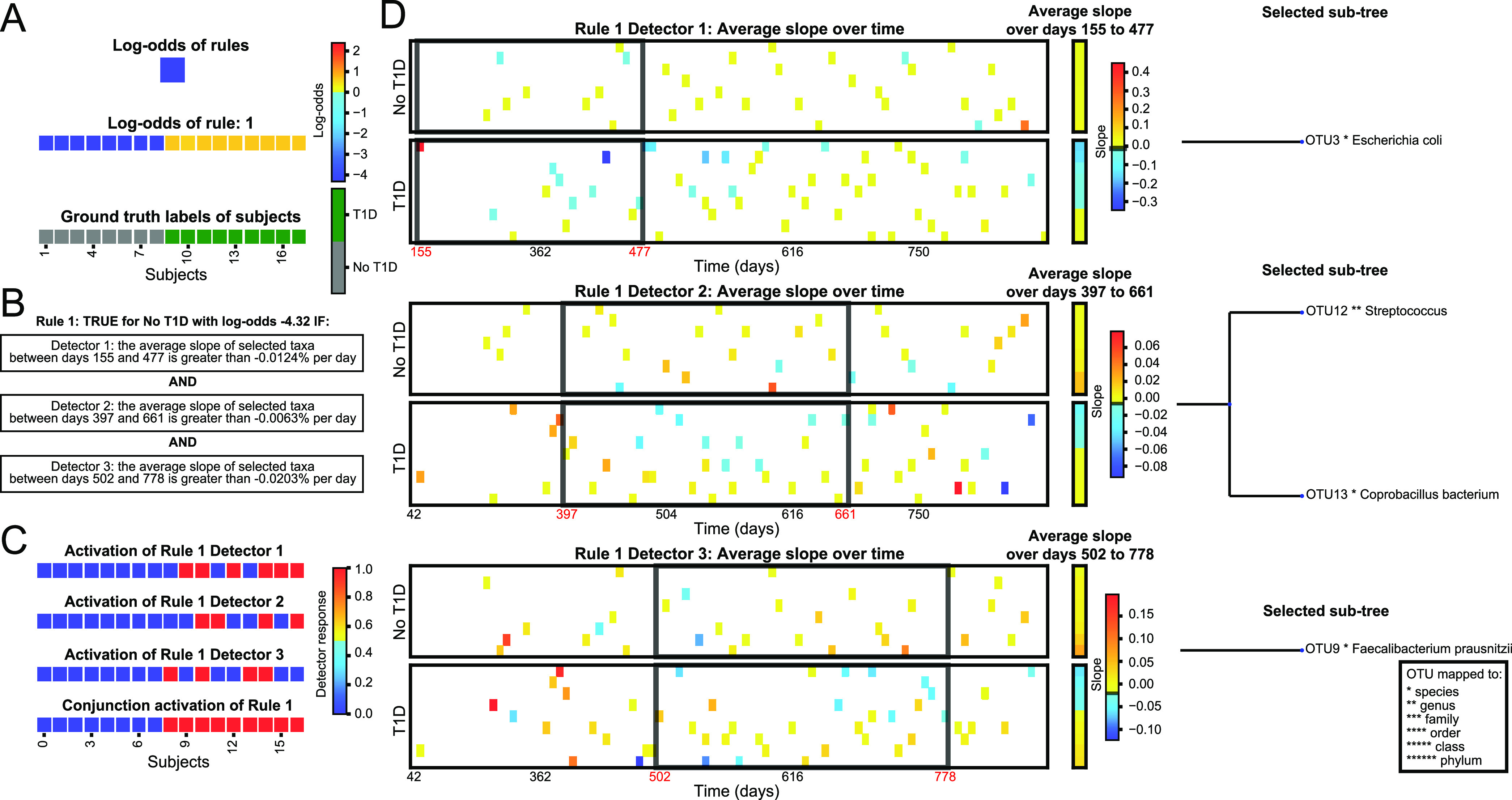
Case study two: MDITRE rules that predict children who developed type 1 diabetes versus those who did not based on a series of gut microbiome changes over time that precede disease onset. Visualizations output by the MDITRE graphical user interface are shown for analysis of data from Kostic et al., who tracked the gut microbiomes of 17 infants during the first 3 years of life using shotgun metagenomics sequencing. (A) MDITRE identified one rule that predicts which children developed type 1 diabetes. Colors indicate the degree to which each rule, or the rules in combination, contribute to the odds of predicting the status (i.e., type 1 diabetes) of each child. Yellow-to-red colors indicate that the child is more likely to develop type 1 diabetes (with red indicating the highest likelihood); cyan-to-blue colors indicate that the child is less likely to develop type 1 diabetes (with blue indicating the least likelihood). (B) Human-readable logic of the rule, which has three detectors. (C) Per-subject activations (“truth” values) for each detector and the conjunction (combined effects) of the detectors. Colors indicate the degree to which each detector (clause) of the rule is true for each child. Red indicates that the detector is true, blue indicates that the detector is false. (D) Visualization of the per-subject data aggregated by each detector, as well as the detector’s window-time (black boxes), threshold for slope (black line on legend), and selected taxa. Note that the detectors identify a pattern of temporal changes that appears to correspond to normal microbiome succession events that are absent or blunted in infants who later develop type 1 diabetes. Colors indicate the degree to which the average aggregated relative abundance (or rate of change in relative abundance) is above or below the threshold of the detector. Yellow-to-red indicates above the threshold (with red the highest value) and cyan-to-blue indicates below the threshold (with blue the lowest value).

**(ii) Case study 2: temporal series of changes in the microbiome preceding onset of type 1 diabetes.** Our second case study used the Kostic et al. data set, which tracked children’s gut microbiomes over the first 3 years of life using shotgun metagenomics, and assessed the onset of type 1 diabetes (T1D). In this case, MDITRE found a single rule (Fig. 5A and B), which contains three detectors of slope-types (Fig. 5C), covering progressive time windows throughout the study: approximately 5 to 15 months, 13 to 22 months, and 17 to 26 months (Fig. 5D). In all cases, each detector was true for increases of the selected taxa, which occurred in children who did not develop T1D. The taxa selected were Escherichia coli for the earliest detector, Streptococcus and *Coprobacillus* for the middle detector, and Faecalibacterium prausnitzii for the latest detector (Fig. 5D). Interestingly, these taxa are progressively anaerobic and specialized to the gut, consistent with community succession events that occur in the normal developing infant gut (38). Thus, the rule MDITRE discovered to classify T1D versus non-T1D infants specifies a temporal pattern of events that appears to detect normal microbiome succession events in healthy infants, which are absent or blunted in infants who later develop T1D.

## DISCUSSION

We have presented MDITRE, software for learning human-interpretable models that predict host status from microbiome time-series data, which achieves comparable predictive performance to our original method in almost all cases, while running up to orders of magnitude faster. Moreover, we have introduced new visualization capabilities and shown through case studies that our method uncovers rich but readily interpretable and biologically relevant patterns in microbiome data sets. To achieve these improvements, we introduced several innovations, including relaxation techniques that operate on temporal or phylogenetic information to yield models that are fully differentiable. With these innovations, we took advantage of standard machine learning libraries that support GPU acceleration and are easily deployable on different operating systems.

We foresee several directions for future work. MDITRE currently supports binary host labels, which we found to be the most common scenario for microbiome data sets. However, the model could readily be extended to multiclass learning. In addition, the model could be extended to time-to-event (survival) prediction tasks, which are relevant for some recent microbiome studies, such as predicting the risk of nosocomial infections (39, 40) and the likelihood of disease resolution (19). Similarly, longitudinal microbiome studies are beginning to include multiple data modalities (41). Due to its layered nature, MDITRE can be extended to incorporate additional multiomics data, such as metabolomics or transcriptomics information. Finally, although MDITRE is a Bayesian model, we used a maximum *a posteriori* inference procedure that cannot estimate uncertainty throughout the model. Future work could involve applying inference techniques, such as Variational Inference (42) or Hamiltonian Monte-Carlo (43), which take advantage of MDITRE’s differentiability while also estimating the posterior distribution.

A limitation of our findings, and indeed an overall shortcoming in the microbiome field, is the extent to which true generalization performance can be demonstrated for statistical and machine learning methods. We used several strategies to mitigate this limitation, including generating independent test sets with different noise characteristics for simulated data, repeated cross-fold validation for six real data sets, and true hold-out validation for one real data set. However, the gold standard for generalization performance would be testing on independent studies (e.g., carried out by separate groups of investigators in different geographic regions) with comparable experimental designs. To our knowledge, no such relevant longitudinal microbiome studies exist, highlighting an important gap in the field that warrants attention.

Overall, we have introduced MDITRE, a new software package that improves on our prior work with orders of magnitude faster run-times and expanded visualization capabilities, to address an important gap in the field: linking changes in the microbiome over time to the status of the host. Benchmarking on semi-synthetic and real data sets shows that our software performs on par with or outperforms a high-capacity interpretable machine learning method (random forests) in almost all cases, in terms of predictive performance, while returning human-interpretable rules that capture domain-specific features of microbiome time-series data. We believe that MDITRE will provide a valuable tool for the microbiome research community, fostering new insights into how changes in the microbiome over time maintain health or lead to disease in humans and other species.

## MATERIALS AND METHODS

### MDITRE model.

We describe the model in terms of a five-layer Bayesian neural-type architecture: (1) phylogenetic focus, (2) temporal focus, (3) detectors, (4) rules, and (5) classification. Text S1 provides an alternate view, as a graphical (plate) model showing the probabilistic structure of MDITRE. The model is fully differentiable, and we implemented maximum *a posteriori* (MAP) inference using gradient-descent.

**(i) Phylogenetic focus layer.** This layer learns localized features by aggregating abundances of phylogenetically related OTUs. Let $\text{P}\in R^{N\times N}$ denote a dissimilarity matrix, e.g., pairwise phylogenetic distances. To allow differentiability, we embed *P* into a *D-dimensional* space using Principal Coordinate Analysis. Our software automatically chooses the value of *D* for each data set by running a two-sample Kolmogorov-Smirnov (KS)-test using the original phylogenetic distances and the distances after embedding, for *D* values ranging from 1 to 30, and choosing the lowest value of *D* with a *P*-value > 0.05 (the lowest number of dimensions such that the original and embedded distributions of distances are not significantly different). Let $E\in R^{N\times D}$ denote the resulting embedding matrix. We assume that each detector *j* in rule *k* has a phylogenetic “center”${\text{γ}}_{\mathrm{kj}}\in R^{D}$ and scalar radius κ_*kj*_; we assume Normal and Lognormal priors on γ_*kj*_ and κ_*kj*_, respectively.

The distance $\xi_{\mathrm{kji}}$ between detector *j*’s phylogenetic center (in rule *k*) and OTU *i*’s phylogenetic embedding is defined as:

(1) $$\xi_{\mathrm{kji}}={\left|\left|{\text{γ}}_{\mathrm{kj}}\;-\;E_{i}\right|\right|}_{2}$$

The layer’s output *a_skj_*, for each host *s*, is then a “soft” aggregation of OTUs that fall within detectors’ phylogenetic centers:

(2) $$u_{\mathrm{kji}}=\text{sigmoid}\left(\left(\kappa_{\mathrm{kj}}-\xi_{\mathrm{kji}}\right)/\tau_{u}\right)$$

(3) $$a_{\mathrm{skj}}=\overset{\text{N}}{\underset{\text{i}}{\sum }}u_{\mathrm{kji}}X_{\mathrm{si}}$$

Here, *X_sit_* denotes a microbiome measurement for time-series *s* at time *t* for OTU *i* of an *N*-dimensional data source (e.g., relative abundances of OTUs from 16S rRNA amplicon or metagenomic shotgun sequencing). The parameter $\tau_{u}$ is a temperature parameter that increases the sharpness of focus with lower temperatures.

We place priors on the phylogenetic centers and radii to encourage focus on interpretable regions of the tree. For κ, we use a Normal prior with mean set to the median of all family-level distances of OTUs and variance set to the median of all variances of family-level distances of OTUs, both of which are derived from the embedding of the reference phylogenetic tree. See Supplemental Methods for complete details.

**(ii) Temporal focus layer.** This layer models two types of localized features in the temporal space: (1) average abundances over a time window, or (2) rates of change (slope) of abundances over a time window.

*Average abundances over time windows*: we use a similar approach to that described for the phylogenetic focus layer. Each detector probabilistically selects a time-window center and extent, and then data from time points in that window are “softly” averaged. Let μ_kj_ denote the time-window center for detector *j* in rule *k* and σ_*kj*_ its corresponding extent (length). We model the random variables μ_*kj*_ and σ_*kj*_ in terms of the fraction of the total experiment length; see Supplemental Methods for complete details.

The output of this layer, *b_skj_*, for each host *s*, is then a (soft) average over the phylogenetically focused microbial abundance data from the previous layer:

(4) $$b_{\mathrm{skj}}=\overset{\text{T}}{\underset{\text{t}=0}{\sum }}a_{\mathrm{skjt}}v_{\mathrm{kjt}}$$

(5) $$v_{\mathrm{kjt}}=\frac{h_{\mathrm{kjt}}}{\sum_{\text{t}=0}^{\text{T}}h_{\mathrm{kjt}}}$$

Here, *h_kjt_* are (soft) indicators as to whether time points occur in the time window. We compute *h_kjt_* as follows, using a relaxed approximation to the Heaviside boxcar function with temperature τ_*v*_.

(6) $$h_{\mathrm{kjt}}=\text{sigmoid}\left(\left(\text{t}-{\text{μ}}_{\mathrm{kj}}+{\text{σ}}_{\mathrm{kj}}/2\right)/\tau_{v}\right)-\text{sigmoid}\left(\left(\text{t}-{\text{μ}}_{\mathrm{kj}}-{\text{σ}}_{\mathrm{kj}}/2\right)/\tau_{v}\right)$$

*Rate of change of abundances (slopes) over time windows*: we use a similar approach as for average abundances over time windows, described above. Briefly, we (softly) estimate the slope over the time window using weighted Ordinary Least Squares (OLS) regression with weights $v_{\mathrm{kjt}}^{\text{′}}$ computed as for average abundances. See Supplemental Methods for complete details.

We set priors on random variables in the temporal focus layer to encourage time windows that correspond to intervals of time relevant to the underlying studies’ experimental designs. These settings encode prior beliefs that time windows encompass 30% of the total study duration and are centered at the midpoint of the study. However, we intend for these priors to be relatively weak, and thus set large variances to create diffuse priors.

**(iii) Detector layer.** The detector layer takes its inputs from the previous layers, which consist of phylogenetically and temporally focused features, and computes activations according to whether the feature is above learned thresholds. By design, the detectors effectively form human-interpretable clauses, i.e., for detector *j* in rule *k*, “*TRUE if* *b_skj_* *(the [aggregated abundance/rate of change of abundance] of organisms within the detectors’ phylogenetic radius and time window) is above threshold* *η_kj_*.” To maintain differentiability, we model the activations using sigmoidal responses with a temperature parameter that is annealed toward increasing sharpness of detectors; we place uniform priors on the threshold random variables. We set the maximum number of detectors per rule to 10, based on our prior work (17), where we showed this to be a very liberal setting for microbiome data sets (e.g., most rules contained only a few detectors).

**(iv) Rule layer.** The rule layer takes the detector activations as inputs and performs a relaxed logical conjugation. We use a relaxation inspired by arithmetic-operation learning networks, which has less impact from vanishing gradients (27) than an exponentiation-based relaxation. The activation for rule *k* for time-series *s* is thus:

(7) $$r_{\mathrm{sk}}=\overset{\text{J}}{\underset{\text{j}=0}{\prod }}\left(1-z_{\mathrm{kj}}\left(1-g_{\mathrm{skj}}\right)\right)$$

Here, *J* is the maximum number of detectors per rule. The random variables z_kj_ (softly) select which detectors are relevant to each rule, using a sigmoid function applied to Normally distributed latent variables; see Supplemental Methods for complete details.

To encourage model parsimony, we place Negative Binomial priors on the number of detectors per rule $\overset{\text{M}}{\underset{\text{j}=0}{\sum }}z_{\mathrm{kj}}$, parameterized by mean *θ_z_* and variance $\theta_{\text{z}}^{\prime }$:

(8) $$\overset{\text{M}}{\underset{\text{j}=0}{\sum }}z_{\mathrm{kj}}\sim \text{NegativeBinomial}\left(\theta_{\text{z}},\theta_{\text{z}}^{\prime }\right)$$

For the data sets analyzed, we set $\theta_{\text{z}}=1$ and $\theta_{\text{z}}^{\prime }=5$, encoding a prior that the 98th percentile of this distribution equates to 50% of model capacity (≤5 active detectors per rule). We set the maximum number of rules to 10, based on our prior work (17), where we showed this to be a very liberal setting for microbiome data sets (e.g., most predictors had fewer than three rules).

**(v) Classification layer.** The classification layer takes the rules’ activations and combines them via a logistic regression model to predict the binary label for each subject. We place diffuse priors on the regression coefficients. Analogous to the detector layer, we introduce random variables *q_k_* that (softly) select which rules are relevant to the final predictor; we similarly place a Negative Binomial prior on the total number of rules in the model, to encourage parsimony. As with the detector layer, we set the mean of this prior to 1 and the variance to 5. See Supplemental Methods for complete details.

### Initialization.

To initialize the phylogenetic focus layer centers and radii, we performed *K*-means clustering on the OTUs in the phylogenetic embedding space (with *K* equal to the maximum number of detectors per rule, *J*), and then set the initial detector phylogenetic centers and radii from the *K*-means output. To initialize the temporal focus layer time-window centers and durations, we set initial values to randomly selected time windows from *N_w_* consecutive segments of the total experiment duration. We compute *N_w_* to be the maximum number of consecutive time intervals while ensuring the presence of at least 2 samples per subject in each interval. The procedure is explained in the algorithm below for initialize detector time windows and centers. The inputs are experimental duration (*T*) and number of time intervals (*N*_w_).

**Algorithm 1:** Initialize detector time-windows and centers

**Input:** Experiment duration *T*, Number of time intervals *N_w_*

1. Divide the experiment duration into *N_w_* equal time windows *T*_0_, *T*_1_, … , *T*_*Nw*_

2. **For** rule *k*, detector *j* **do**

3.  Randomly choose a time window *t*_*init*_ from *T*_0_, *T*_1_, … , *T*_*Nw*_

4.  Initialize μ_*kj*_ to midpoint of *t*_*init*_

5.  Initialize σ_*kj*_ to length of *t*_*init*_

6. **End for**

To initialize the detector layer abundance or slope thresholds, we set the initial values equal to the mean over all subjects of the aggregated abundances (or slopes) computed based on the initializations of the phylogenetic or temporal focus parameters, as described above. We initialized logistic regression coefficients from a standard normal distribution and set initial bias terms to zero. We initialized the rule and detector selectors to 0.5, which ensures equal probability of a rule or detector being active at the start of the training.

### Model training and testing.

We performed MAP inference using RMSProp. We used a learning rate of 0.01 for the temporal focus layer parameters (μ, σ) and 0.001 for all other parameters. We found that in our experiments, the temporal focus parameters needed a higher learning rate based on their scale, which is typically an order of magnitude higher than all the other parameters. For example, the temporal focus parameters typically are on the order of 10 to 100 days, whereas other parameters such as rule and detector selectors, thresholds, and phylogenetic windows are typically on the order of 0 to 1. For full learning rate settings, please refer to our code, available from https://github.com/gerberlab/mditre.

Temperature parameters were linearly annealed throughout learning toward sharpness. The temperature parameters for phylogenetic, temporal, rule and classification layers were annealed from 1 to 0.1. The temperatures for thresholds for detectors were annealed from 10^–2^ to 10^–3^ and from 10^–3^ to 10^–4^ for aggregated abundances and slopes, respectively; these ranges correspond to the scales of abundances or slopes in data. The model was trained using the RMSprop optimizer (with default parameters, as given here: https://pytorch.org/docs/stable/generated/torch.optim.RMSprop.html#torch.optim.RMSprop) for 2,000 iterations on all the data sets. We determined model convergence by defining a stopping criterion, which halts the training process once the training loss stops decreasing (within a numerical tolerance) within a window of training iterations. Specifically, the training process was stopped once the training loss did not decrease by at least 1 unit of loss within the last 100 iterations. See also Fig. S6, which provides plots of the training loss on each data set over the iterations.

10.1128/msystems.00132-22.6 FIG S6 Training loss plots for MDITRE on real data. Download FIG S6, TIF file, 0.1 MB. Copyright © 2022 Maringanti et al. 2022 Maringanti et al. https://creativecommons.org/licenses/by/4.0/ This content is distributed under the terms of the Creative Commons Attribution 4.0 International license.

We used the following hardware configuration for benchmarking: an Intel Xeon Silver 4116 CPU (2.1 GHz) with 24 cores, 48 GB RAM, and an NVIDIA Tesla V100 GPU.

### Performance evaluation on semi-synthetic data.

The data sets used in the semi-synthetic data experiments were generated using MITRE’s synthetic data generation procedure available at https://github.com/gerberlab/mitre/tree/master/mitre (see also our previous work [17]). For the cases with increasing noise levels, data sets were generated by varying the “data_std_percent” parameter (using values 0.3, 3, 30, 300, and 3,000) in MITRE’s data generation model (see MITRE main manuscript [17] and MITRE code repository at https://github.com/gerberlab/mitre/tree/master/mitre).

Model performance for the semi-synthetic data were estimated using a 5-fold cross-validation procedure for model selection followed by validation on an independently generated held-out test data set of the same size as the training data set. This process was repeated 10 times, each with a different random seed. Model classification performance was evaluated using the F1 score (harmonic mean of precision and recall) on the validation data sets and the Mann-Whitney U-test was used for significance testing. In addition, we also evaluated model performance using the Area under curve (AUC) score estimated from the Receiver operating characteristic (ROC) curve on the validation data sets and Delong’s method for significance testing.

### Performance evaluation on real data.

Model performance on all real data sets except for Shao et al. was assessed using 5-fold repeated cross-validation with five repeats (random partitions of the data). For the Shao et al. data set, we assessed classification performance on a test set consisting of 25% of the data (randomly selected), with the remaining 75% used solely for training and model selection. All models were run 10 times, each time using a different random seed. Model performance was evaluated analogously to the semi-synthetic data, using F1 and AUC scores.

### Details on code, implementation, and availability.

The model was implemented in Python 3.6 and PyTorch 1.6 (28). The NumPy 1.21, Scikit-learn 0.24, Matplotlib 3.4, SciPy 1.6, and ETE3 3.1 libraries were also used. The full source code and documentation is available at https://github.com/gerberlab/mditre under a GPL 3.0 license. Scripts to reproduce all results in the manuscript are available at https://github.com/gerberlab/mditre/tree/master/mditre_paper_results. We also provide a tutorial at https://github.com/gerberlab/mditre#usage which describes how to perform tasks such as data loading and preprocessing, running the model, and exploring the output through the graphical user interface.

### Real microbiome data sets and bioinformatics.

The data sets corresponding to Bokulich et al., David et al., Vatanen et al., Digiulio et al., and Kostic et al. were all downloaded as Python pickle objects from https://github.com/gerberlab/mitre/tree/master/mitre. These were input into MDITRE without any further preprocessing. The data sets for Brooks et al. and Shao et al. were first downloaded from the R Bioconductor Package (https://bioconductor.org/packages/release/data/experiment/html/curatedMetagenomicData.html) and then transformed into a Python pickle object and input to MDITRE for modeling. A 5% prevalence cutoff was used to filter taxa to include in the modeling for these two data sets. The phylogenetic prior for 16s rRNA data sets were calculated using a reference phylogenetic tree with 7,500 OTUs, available from https://github.com/gerberlab/MDSINE2_Paper/blob/master/analysis/files/phylogenetic_placement_OTUs/phylogenetic_tree_full.nhx. Placements of OTUs on the tree was performed by running the software pplacer (44) (https://matsen.fhcrc.org/pplacer/). For the metagenomics data sets, phylogenetic distances between taxa were determined after mapping the taxa to a reference tree of 9,700 strains available within the Metaphlan2 package (45) (https://github.com/biobakery/MetaPhlAn/blob/master/metaphlan/utils/mpa_v30_CHOCOPhlAn_201901_species_tree.nwk).

### Data availability.

The data sets supporting the conclusions of this article are available in the following repositories. Bokulich et al. (2) is available in the European Nucleotide Archive (ENA) under accession no. PRJEB14529; David et al. (6) is available in the MG-RAST archive (MG-RAST mgp6248); DiGiulio et al. (30) is available in the Sequence Read Archive (SRA) at PRJNA288562; Kostic et al. (4) is available under SRA: PRJNA231909; Vatanen et al. (31) is available under SRA: PRJNA290380; Brooks et al. (32) is available under SRA: PRJNA376580; Shao et al. (33) is available from ENA ERP115334 and ERP024601. Details on the MDITRE software package are as follows: project name: MDITRE; project home page: https://github.com/gerberlab/mditre; archived version: https://zenodo.org/record/5796297; operating system(s): platform-independent; programming languages: Python; other requirements: Python 3.6 or higher (will run on a CPU, but a GPU provides additional acceleration); license: GPL 3.0. There are no restrictions for use by non-academics.

10.1128/msystems.00132-22.8 TEXT S1 Full mathematical details of the MDITRE probabilistic model. Download Text S1, PDF file, 0.5 MB. Copyright © 2022 Maringanti et al. 2022 Maringanti et al. https://creativecommons.org/licenses/by/4.0/ This content is distributed under the terms of the Creative Commons Attribution 4.0 International license.
