# Supplementary figures and images for: MDITRE: Scalable and Interpretable Machine Learning for Predicting Host Status from Temporal Microbiome Dynamics
Source: mSystems. 2022 Sep 7;7(5):e00132-22. doi: 10.1128/msystems.00132-22 (PMC9600536; doi:10.1128/msystems.00132-22)

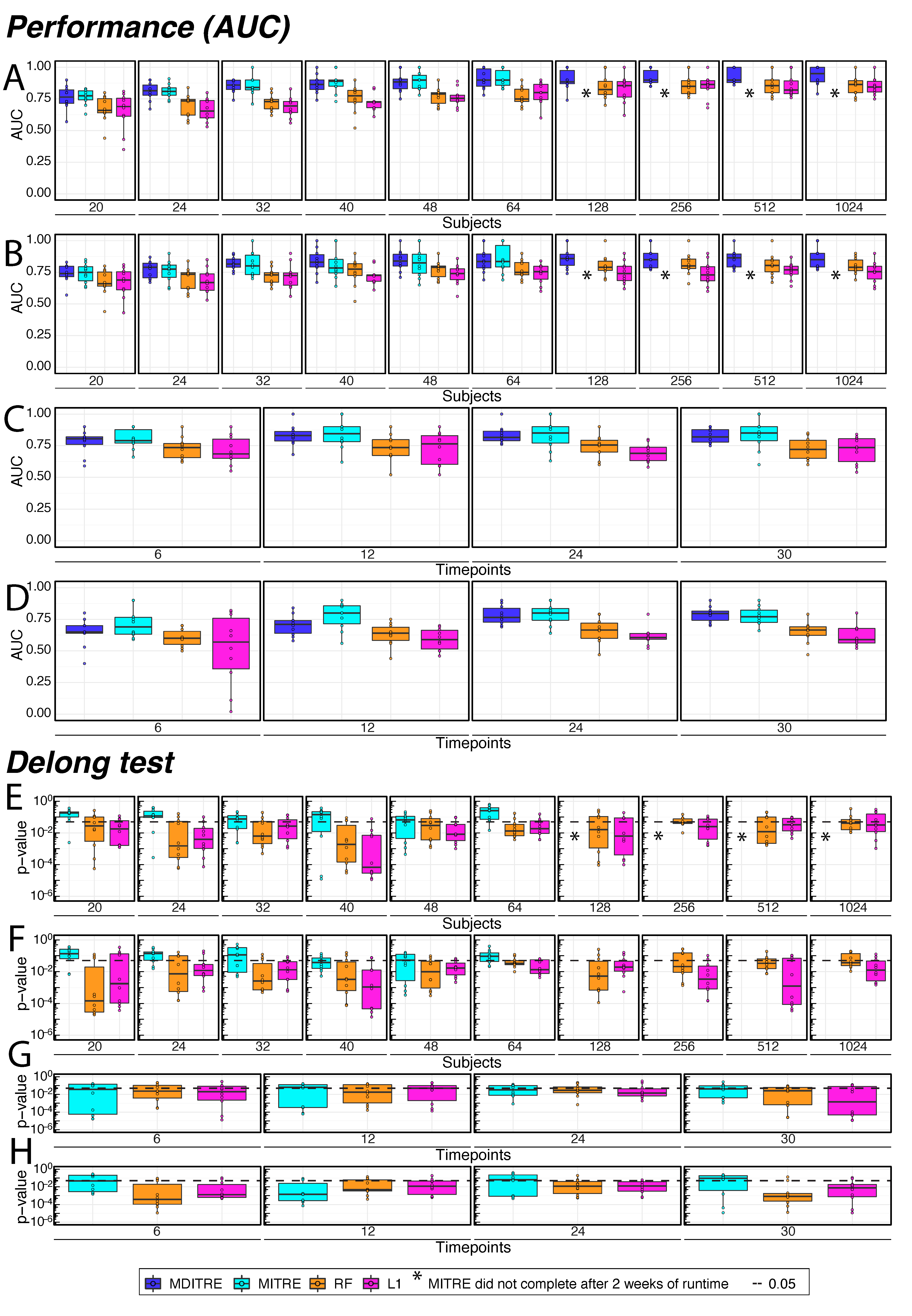

Supplement: FIG S1 [file msystems.00132-22-s0003.tif]

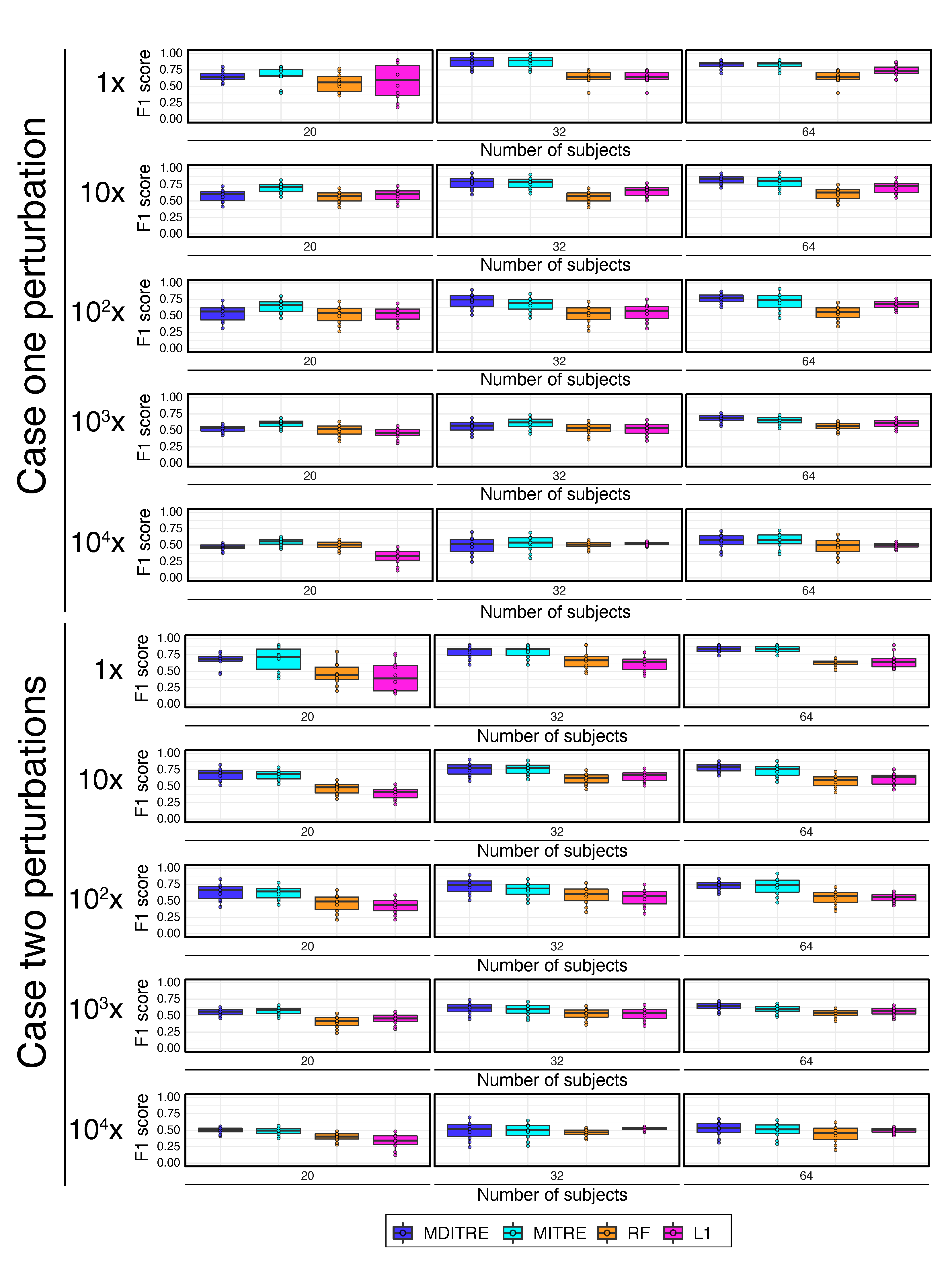

Supplement: FIG S2 [file msystems.00132-22-s0004.tif]

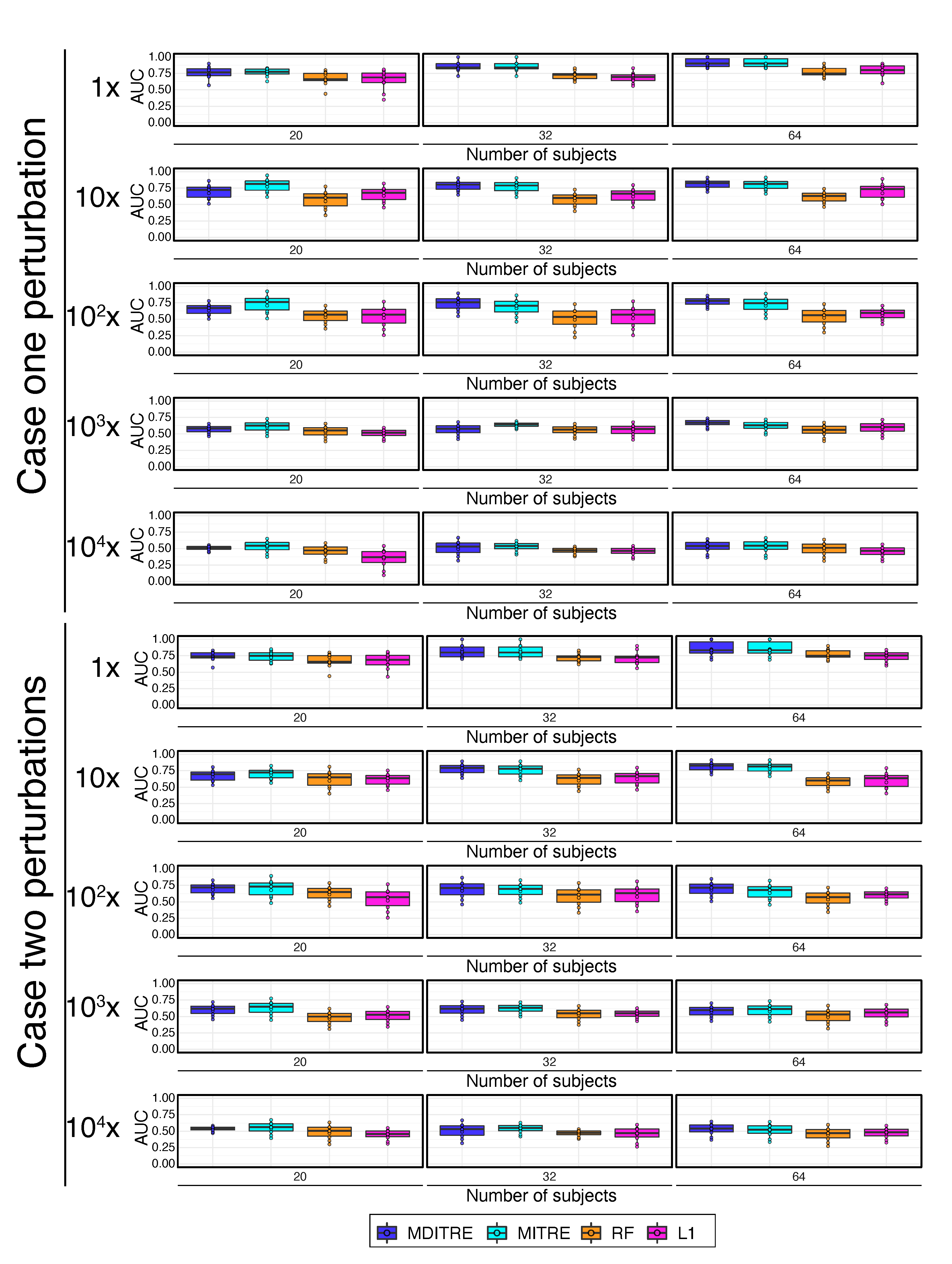

Supplement: FIG S3 [file msystems.00132-22-s0005.tif]

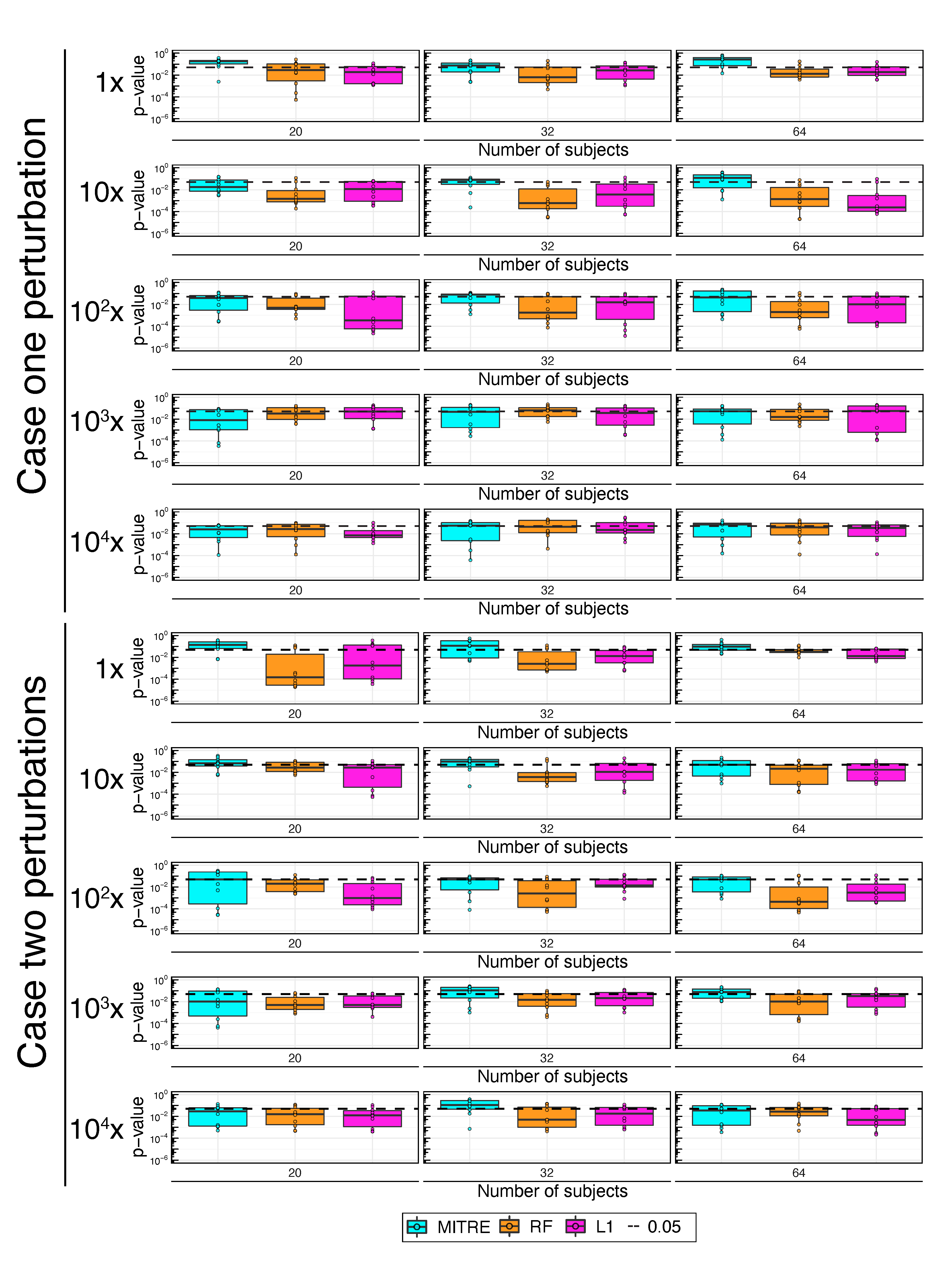

Supplement: FIG S4 [file msystems.00132-22-s0006.tif]

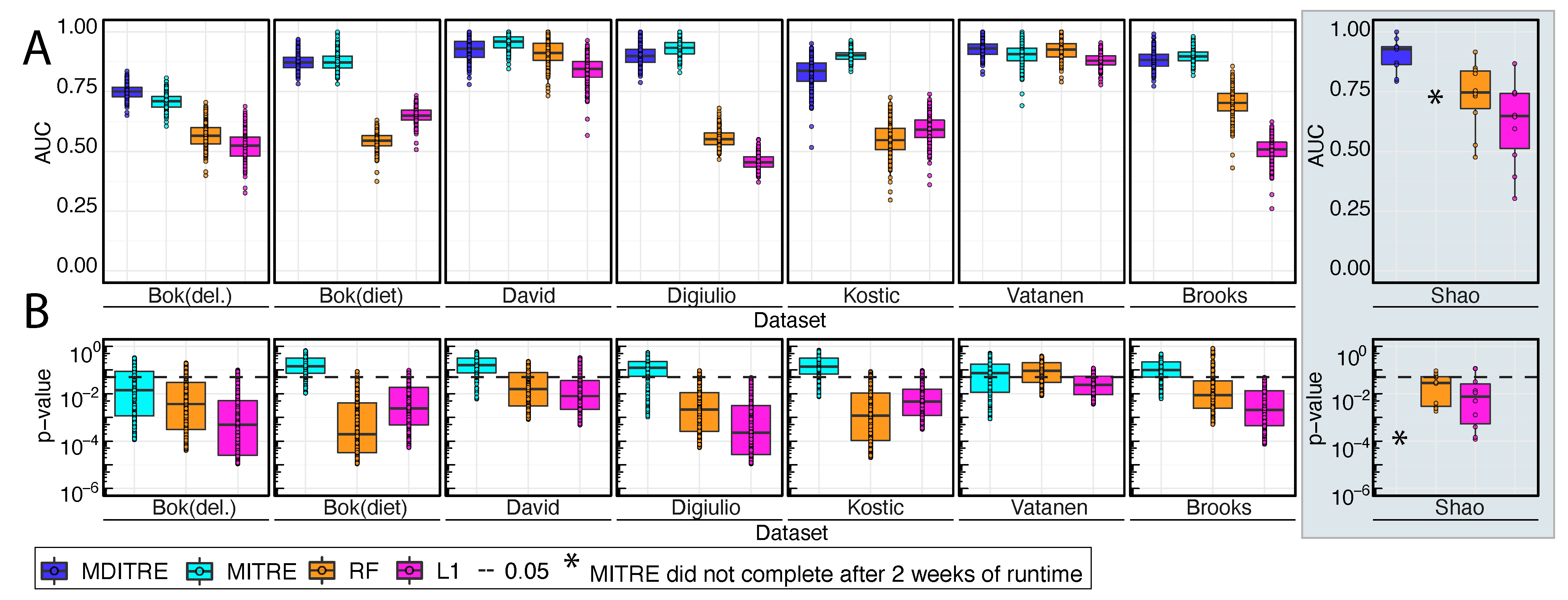

Supplement: FIG S5 [file msystems.00132-22-s0007.tif]

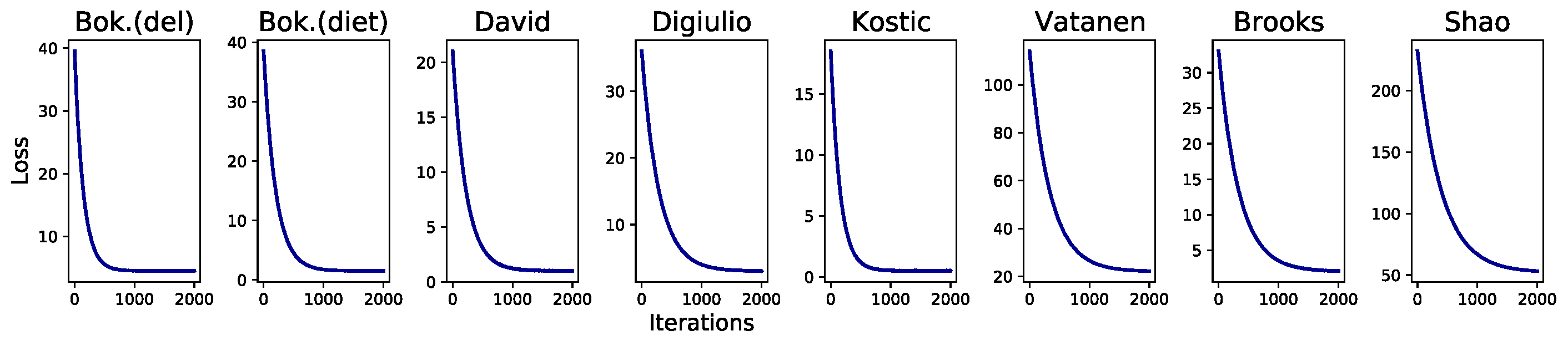

Supplement: FIG S6 [file msystems.00132-22-s0008.tif]
