# Supplementary material for: MDITRE: Scalable and Interpretable Machine Learning for Predicting Host Status from Temporal Microbiome Dynamics
Source: mSystems. 2022 Sep 7;7(5):e00132-22. doi: 10.1128/msystems.00132-22 (PMC9600536; doi:10.1128/msystems.00132-22)
Supplement: TEXT S1 [file msystems.00132-22-s0001.pdf]

MDITRE Supplementary Methods

1 Probabilistic Model

In this section we describe the mathematical details of the MDITRE probabilistic model. Figure 1 provides a graphical model depiction. As described in the main text, the MDITRE model can also be viewed as a five-layer neural-type architecture. Each layer learns relevant features that form the interpretable rules, which are combined in the final layer to predict the class label. The layers are as follows:

- **Phylogenetic focus layer:** Learns relevant regions in an embedded space, and outputs aggregate abundances of phylogenetically similar OTUs.
- **Temporal focus layer:** Learns relevant time-windows, and outputs average abundances or rates of change over selected time-windows.
- **Detector response layer:** Learns thresholds (for abundances or rates of change of abundances) to and outputs (soft) truth values of clauses.
- **Rule response layer:** Learns which clauses (detectors) are relevant and outputs (soft) logical conjunctions of detector outputs.
- **Class prediction layer:** Learns which rules are relevant and how to weight relevant rules, and outputs class label predictions.

1.1 Phylogenetic Focus Layer

This layer learns localized features by aggregating abundances of phylogenetically similar Operational Taxonomic Units (OTUs). Let X_{sit} denote a microbiome measurement from subject s at time t for OTU i of an N-dimensional data source (e.g., relative abundances of OTUs from 16S rRNA amplicon or metagenomic shotgun sequencing). Let $P \in \mathbb{R}^{N\times N}$ denote a phylogenetic distance matrix. We embed P into a latent space of dimensionality D (e.g., via PCoA on P) and denote the resulting embedding matrix as $E \in \mathbb{R}^{N\times D}$. We associate with each rule k, detector centers $\gamma_{kj} \in \mathbb{R}^D$ and scalar radii κ_{kj}, where $j \in \{1, \ldots, J\}$ (M detectors per rule).

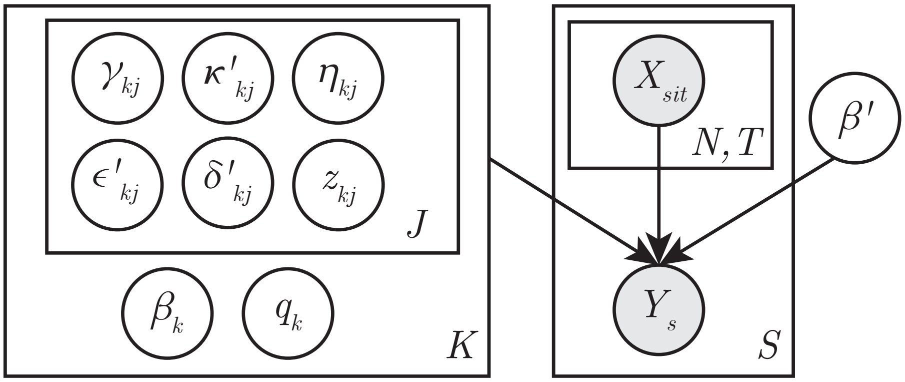

Figure 1: Graphical model depiction of the MDITRE probabilistic model. Observed variables, X_{sit} (microbiome time-series) and Y_s (class labels) are shaded. The nested plates on the left contain latent variables that model K rules and their associated detectors (M detectors per rule). The class label Y_s is predicted via logistic regression on the rules with weights β. The variables z_{kj} and q_k select which detectors or rules are active, respectively. Latent variables have prior probability distributions (not shown; see text for details).

We define the distance d_{kji} between the detector center j in rule k and OTU i's phylogenetic embedding as:

$$\xi_{kji} = \sqrt{\sum_{d}^{D}(\gamma_{kjd} - E_{id})^2} \tag{1}$$

OTUs with distances within radius κ_{kj} are then softly selected for inclusion:

$$u_{kji} = \text{logistic}((\kappa_{kj} - \xi_{kji})/\tau_u) \tag{2}$$

Here, τ_u is a temperature parameter that determines the selection sharpness.

The outputs of the layer are aggregated abundances, a_{skj}, of softly selected OTUs, for each detector j in rule k in subject s:

$$a_{skj} = \sum_{i}^{N} u_{kji} X_{si} \tag{3}$$

We place Normal priors on the detector centers γ_{kj}:

$$\gamma_{kj} \sim \text{MultivariateNormal}(\theta_\gamma, \theta_\gamma^2 I) \tag{4}$$

We place Lognormal priors on the detector radii κ_{kj}:

$$\kappa'_{kj} \sim \quad \text{Normal}(\theta_\kappa, (\theta'_\kappa)^2) \tag{5}$$

$$\kappa_{kj} = \qquad e^{\kappa'_{kj}} \tag{6}$$

$$\tag{7}$$

1.2 Temporal Focus Layer

This layer learns two types of temporally localized features: (1) average abundances b_{skj} over time-windows, and (2) average rates of change of abundances (slopes) b'_{skj} over time-windows.

Relevant time-windows are learned using a temporal focus mechanism that (softly) selects time-points. Each detector j in rule k learns a window center μ_{kj} and duration σ_{kj}. We parameterize σ_{kj} in terms of fractions ϵ_{kj} of the total experimental duration T:

$$\epsilon'_{kj} \sim \text{Normal}(\theta_\epsilon, (\theta'_\epsilon)^2) \tag{8}$$

$$\epsilon_{kj} = \text{logistic}(\epsilon'_{kj}) \tag{9}$$

$$\sigma_{kj} = T\epsilon_{kj} \tag{10}$$

Note that the prior on ϵ'_{kj} specified in Equation 9 can be used to encode knowledge or beliefs about relevant time-window durations.

We parameterize the window centers μ_{kj} similarly:

$$\delta'_{kj} \sim \text{Normal}(\theta_\delta, (\theta'_\delta)^2) \tag{11}$$

$$\delta_{kj} = \text{logistic}(\delta'_{kj}) \tag{12}$$

$$\mu_{kj} = T\epsilon_{kj}/2 + T(1-\epsilon_{kj})\delta_{kj} \tag{13}$$

We use a relaxed boxcar function to compute (soft) selections v_{kjt}, for each time-point t:

$$h_{kjt} = \text{logistic}((t-\mu_{kj}+\sigma_{kj}/2)/\tau_v) - \text{logistic}((t-\mu_{kj}-\sigma_{kj}/2)/\tau_v) \tag{14}$$

$$v_{kjt} = \frac{h_{kjt}}{\sum_t^T h_{kjt}} \tag{15}$$

Here, τ_v is a temperature parameter controlling the sharpness of the approximation to the boxcar function.

1.2.1 Average abundances over time-windows

The output is a (soft) average over the selected time-windows of abundances a_{skjt} (from the previous layer):

$$b_{skj} = \sum_t^T a_{skjt} v_{kjt} \tag{16}$$

1.2.2 Average rates of change of abundances (slopes) over time-window

The output is a (soft) slope over the selected time-windows of abundances a_{skjt} (from the previous layer):

$$b'_{skj} = \frac{(\sum v_{kjt} a_{skit})(\sum v_{kjt} c_{kjt}) - (\sum v_{kjt})(\sum v_{kjt} a_{skjt} c_{kjt})}{(\sum v_{kjt} c_{kjt})^2 - (\sum v'_{kjt} c^2_{kjt})(\sum v'_{kjt})} \tag{17}$$

Here, $c_{kjt} = t - \mu_{kj}$. Note that Equations 17 is the solution to the Weighted Linear Regression problem with weights v_{kjt}.

1.3 Detector Activation Layer

This layer learns activation thresholds η_{kj} and η'_{kj} for each detector j in rule k, i.e., if the input (the phylogenetically and temporally focused abundance b_{skj} [or slope b'_{skj}]) is greater than η_{kj} (or η'_{kj}, the detector is (softly) true. We place a Uniform prior on η_{kj} and η'_{kj}:

$$\eta_{kj} \sim \text{Uniform}(\theta_\eta, \theta'_\eta) \tag{18}$$

$$\eta'_{kj} \sim \text{Uniform}(\theta_\eta, \theta'_\eta) \tag{19}$$

The output of the layer is given by:

$$g_{skj} = \text{logistic}((\eta_{kj} - b_{skj})/\tau_g) \tag{20}$$

$$g'_{skj} = \text{logistic}((\eta'_{kj} - b'_{skj})/\tau_g) \tag{21}$$

Here, τ_g is a temperature parameter that controls the sharpness of the activation.

1.4 Rule Activation Layer

This layer learns which detectors are relevant and then outputs a (soft) logical conjunction of the selected detectors. We model the joint probability of detector selectors as:

$$\begin{aligned} P(z_{k1}, \ldots, z_{kJ}) = \frac{1}{C(\theta_z, \theta'_z)} \prod_j \text{BinaryConcrete}(z_{kj};\ \alpha_z, \tau_z) \\ \times \text{NegativeBinomial}(\sum_j z_{kj};\ \theta_z, (\theta'_z)^2) \end{aligned} \tag{22}$$

Here, each $z_{kj} \in (0, 1)$ and τ_z is a temperature parameter for the BinaryConcrete distribution, which is a continuous relaxation of the discrete Bernoulli distribution. Note that we do not need to compute the normalization constant, $C(\theta_z, \theta'_z)$, because θ_z and θ'_z are fixed parameters that we are not optimizing over.

We use the following PDF of the BinaryConcrete parameterized by location $\alpha_z \in (0, \infty)$ and temperature τ_z:

$$P(X = x; \alpha_z, \tau_z) = \frac{\tau_z \alpha_z x^{-\tau_z - 1}(1 - x)^{-\tau_z - 1}}{(\alpha_z x^{-\tau_z} + (1 - x)^{-\tau_z})^2} \tag{23}$$

Equation 22 also places a prior probability distribution on the total number of active detectors in each rule through the NegativeBinomial distribution on the sum of detector selector variables. We use this distribution to encode model sparsity (inductive bias toward small numbers of detectors).

We use the following PDF for the Negative Binomial, which is parameterized in terms of its mean θ_z and variance $\theta_{z'}$ and allows for non-integer values:

$$P(X = x) = \frac{\Gamma(\frac{\theta_z^2}{(\theta_z')^2 - \theta_z} + x)}{\Gamma(x+1)\Gamma(\frac{\theta_z^2}{(\theta_z')^2 - \theta_z})} \left(\frac{(\theta_z')^2 - \theta_z}{(\theta_z')^2}\right)^x \left(\frac{\theta_z}{(\theta_z')^2}\right)^{\frac{\theta_z^2}{(\theta_z')^2 - \theta_z}} \tag{24}$$

The outputs of the layer are rule activations, r_{sk}, which are relaxed logical conjunctions (modeled as products) of selected detector responses g_{skj}:

$$r_{sk} = \prod_j^M (1 - z_{kj}(1 - g_{skj})) \tag{25}$$

1.5 Prediction Layer

This layer learns which rules are relevant and weights for the rules, which are then used to predict the probabilities of the binary class labels Y_s. We model the (soft) rule selectors, q, analogously to the detector selectors described above:

$$\begin{aligned} P(q_1, \ldots, q_K) = \frac{1}{C(\theta_q, \theta_q')} \prod_k &\text{BinaryConcrete}(q_k;\ \alpha_q, \tau_q) \\ &\times \text{NegativeBinomial}(\sum_k q_k;\ \theta_q, (\theta_q')^2) \end{aligned} \tag{26}$$

Analogous to the prior on detector selectors, Equation 26 places BinaryConcrete priors on the rule selectors and a NegativeBinomial distribution on the total number of active rules in the model. We use the latter to encode model sparsity (inductive bias toward a small number of rules).

We use a logistic regression model over the selected rules to predict class probabilities:

$$\phi_s = \sum_k^K q_k r_{sk} \beta_k + \beta' \tag{27}$$

$$p_s = \text{logistic}(\phi_s) \tag{28}$$

$$Y_s \sim \text{Bernoulli}(p_s) \tag{29}$$

Finally, we place Normal priors on the regression coefficients (weights) β:

$$\beta_k \sim \text{Normal}(0, (\theta_\beta)^2) \tag{30}$$

$$\beta_k' \sim \text{Normal}(0, \theta_{\beta'})^2) \tag{31}$$
